# Involvement of PUF60 in Transcriptional and Post-transcriptional Regulation of Hepatitis B Virus Pregenomic RNA Expression

**DOI:** 10.1038/s41598-017-12497-y

**Published:** 2017-10-09

**Authors:** Suofeng Sun, Kenji Nakashima, Masahiko Ito, Yuan Li, Takeshi Chida, Hirotaka Takahashi, Koichi Watashi, Tatsuya Sawasaki, Takaji Wakita, Tetsuro Suzuki

**Affiliations:** 1 Department of Virology and Parasitology, Hamamatsu University School of Medicine, Shizuoka, 431-3192 Japan; 2 Proteo-Science Center, Ehime University, Ehime, 790-8577 Japan; 3 Department of Virology II, National Institute of Infectious Diseases, Tokyo, 162-8640 Japan

## Abstract

Here we identified PUF60, a splicing factor and a U2 small nuclear ribonucleoprotein auxiliary factor, as a versatile regulator of transcriptional and post-transcriptional steps in expression of hepatitis B virus (HBV) 3.5 kb, precore plus pregenomic RNA. We demonstrate that PUF60 is involved in: 1) up-regulation of core promoter activity through its interaction with transcription factor TCF7L2, 2) promotion of 3.5 kb RNA degradation and 3) suppression of 3.5 kb RNA splicing. When the 1.24-fold HBV genome was introduced into cells with the PUF60-expression plasmid, the 3.5 kb RNA level was higher at days 1–2 post-transfection but declined thereafter in PUF60-expressing cells compared to viral replication control cells. Deletion analyses showed that the second and first RNA recognition motifs (RRMs) within PUF60 are responsible for core promoter activation and RNA degradation, respectively. Expression of PUF60 mutant deleting the first RRM led to higher HBV production. To our knowledge, this is the first to identify a host factor involved in not only positively regulating viral gene expression but also negative regulation of the same viral life cycle. Functional linkage between transcriptional and post-transcriptional controls during viral replication might be involved in mechanisms for intracellular antiviral defense and viral persistence.

## Introduction

Hepatitis B virus (HBV) is a hepatotropic, enveloped virus of the *Hepadnaviridae* family with a partial double-stranded relaxed circular DNA genome. Approximately 240 million people worldwide are chronically infected with HBV. It is estimated that one million deaths occur annually due to HBV-related severe liver diseases such as liver cirrhosis, liver failure and hepatocellular carcinoma^1^. Although nucleoside analogues and interferons are the major chemotherapies for HBV-positive patients to date, they do not achieve HBV clearance or eliminate the viral genome when in the covalently closed circular (ccc) DNA form, which resides in the nucleus of infected cells. Long-term treatment with these antivirals may also have drawbacks such as development of drug-resistant variants and adverse side effects^2,3^. Thus, understanding of molecular mechanisms that determine viral replication, persistence and latency is urgently needed to develop novel treatments to achieve virological cure.

Upon infection, the uncoated viral genome is transported to the nucleus and converted into cccDNA, which serves as the template for synthesis of viral transcripts. Four unspliced viral RNAs, 3.5, 2.4, 2.1 and 0.7 kb, are transcribed from their respective promoters and two enhancer regions (ENI and ENII). The 3.5 kb RNA includes precore and pregenomic RNA species. Precore mRNA encodes precore antigen (HBeAg), and pregenomic RNA directs translation of core antigen (HBcAg) and polymerase. Pregenomic RNA also serves as a reverse transcription template after encapsidation. A variety of liver-enriched and ubiquitous transcription factors target the promoter and enhancer regions to regulate viral transcription and replication (as reviewed in^4,5^). In addition, several forms of spliced RNAs are generated from 3.5 kb RNA. These spliced forms have been observed in sera and livers of hepatitis B patients as well as in cultured cells transfected with the viral genome^6–8^. However, their significance and regulatory mechanisms underlying post-transcriptional processing events in the HBV life cycle are essentially unclear.

In this study, we aimed to clarify molecular mechanisms controlling transcriptional and post-transcriptional processes during HBV replication, in particular mechanistic coupling between transcriptional regulation and post-transcriptional mRNA processing. During the course of investigating involvement of host cell factors with dual DNA- and RNA-binding capacities in HBV replication in siRNA-mediated gene knockdown and over-expression experiments, we identified PUF60 as a versatile regulator of both transcriptional and post-transcriptional steps in expression of HBV 3.5 kb RNA. PUF60 was first discovered as a poly-U binding, 60-kDa splicing factor that is important for efficient splicing of multiple introns^9^. In addition, PUF60 forms a complex with far upstream element (FUSE) and FUSE-binding protein (FBP), acting as an FBP-interacting repressor (FIR), and is a transcriptional repressor of human c-myc gene^10^. Here, we found that PUF60 up-regulated core promoter activity through its interaction with transcription factor 7-like 2 (TCF7L2), which is necessary for direct binding with the ENII region. PUF60 also contributed to 3.5 kb RNA degradation and suppression of 3.5 kb RNA splicing.

## Results

### Involvement of PUF60 in positive and negative regulation on HBV replication

**Figure 1:**
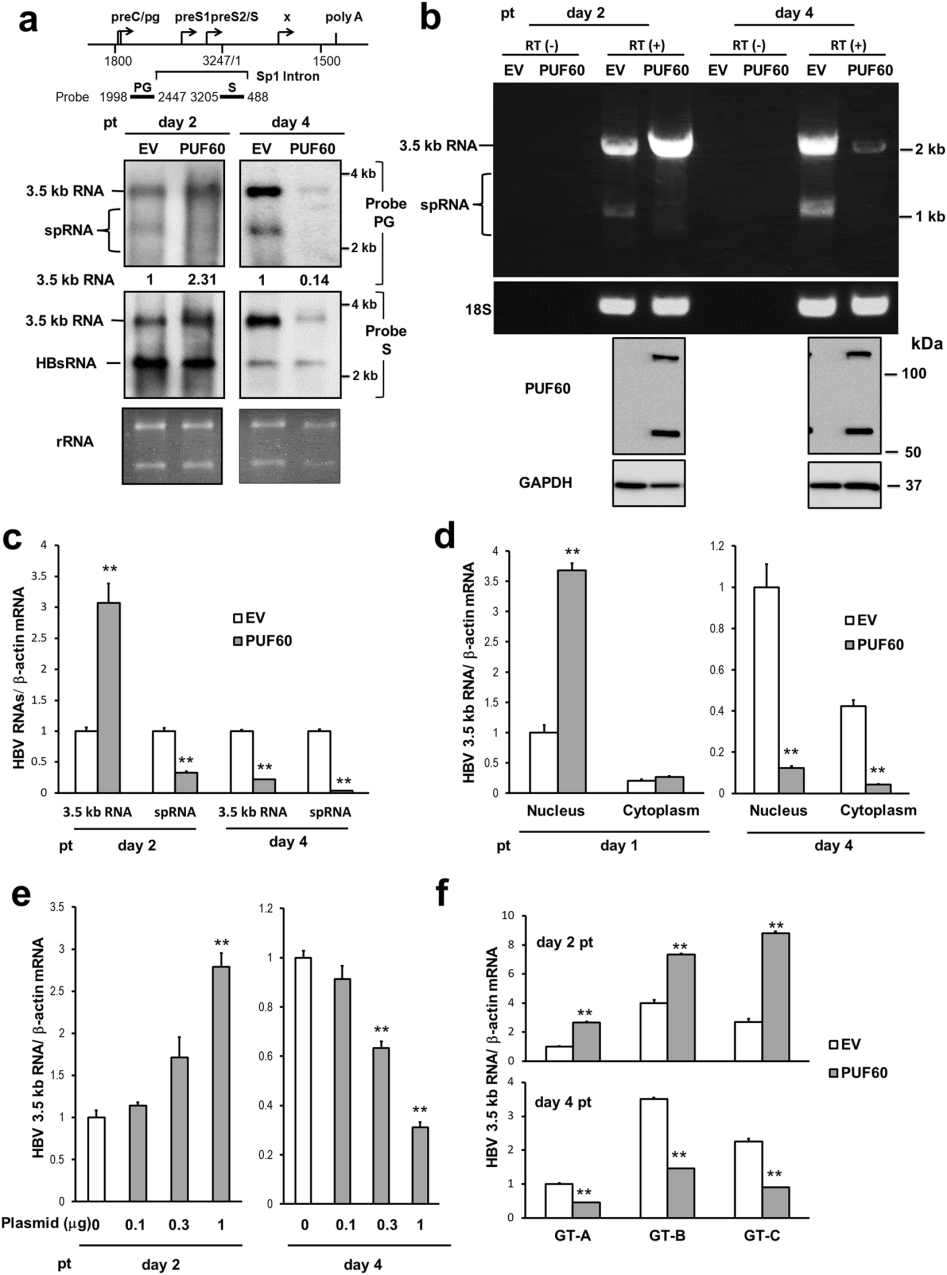
Involvement of PUF60 in regulation of HBV RNA expression. (**a**) A schematic diagram of HBV RNAs and regions used as probes for northern blotting is indicated (top). pcDNA-F-PUF60 or an empty vector (EV) was co-transfected with pUC-HB-Ce into HuH-7 cells. At day 2 or 4 post-transfection (pt), total RNA was extracted from cells and separated on an agarose gel. HBV 3.5 kb RNA and spRNA (upper panels) and 3.5 kb RNA and HBs RNA (lower panels) were detected by northern blotting using probe PG (nt 1998–2447) and probe S (nt 3205–488), respectively. Band intensities of 3.5 kb RNA on the blots with PG probe were determined by Image-J software and those of control samples (EV) were calculated as 1. (**b**) Total RNAs prepared as described above were used for semi-quantitative RT-PCR with (RT(+)) or without (RT(−)) reverse transcription. cDNA bands corresponding to unspliced 3.5 kb RNA and its spliced forms (spRNAs) were detected by agarose gel electrophoresis. 18 S ribosomal RNA (18 S) was also detected. Immunoblotting indicated expression of PUF60 and GAPDH in transfected cells. (**c**) RT-qPCR analysis was performed to determine 3.5 kb RNA and spRNA levels in cells as described above. (**d**) Nuclear and cytoplasmic fractions of cells transfected with pUC-HB-Ce with pcDNA-F-PUF60 or EV were isolated and 3.5 kb RNA levels in each fraction were determined at days 1 and 4 pt. (**e**) Dose-dependent effect of PUF60 on 3.5 kb RNA levels was determined in cells transfected with pUC-HB-Ce with various concentrations of pcDNA-F-PUF60 by RT-qPCR. (**f**) Effect of PUF60 expression on 3.5 kb RNA levels of various HBV genotypes was determined in cells transfected with pcDNA-F-PUF60 and a plasmid carrying the 1.24-fold HBV genome derived from HBV genotype (GT) A, B or C. (**c**)–(**f**) Data are normalized to that of β-actin mRNA and values of “EV” (GT-A EV in case of (**f**)) are set to 1. Values shown represent means ± SD obtained from three independent samples. Statistical differences compared with the control (EV) are shown. **p < 0.01, Student’s t test. Full-length blots in (**a**) and (**b**) are presented in Supplementary Figures S14 and S15, respectively.

First, to address how PUF60 is involved in gene expression and HBV replication, viral RNAs in cells co-transfected with pUC-HB-Ce carrying the 1.24-fold HBV genome derived from genotype C and the FLAG-tagged PUF60-expressing plasmid (pcDNA-F-PUF60) were analyzed by northern blotting. At day 2 post-transcription (pt), the level of 3.5 kb RNA, but not 3.5 kb RNA -derived, 2.2-kb spliced (Sp1) RNA lacking intron nt 2447/489, was higher in PUF60-expressing cells compared to control cells. In contrast, at day 4 pt, both 3.5 kb RNA and Sp1 RNA levels were severely diminished in PUF60-expressing (Fig. 1a, probe PG). Influence of PUF60 expression on the HBs RNA level appeared limited compared to that on 3.5 kb RNA and Sp1 RNA levels (Fig. 1a, probe S). We confirmed that no cytotoxic effect was observed by over-expressing PUF60, as judged by ribosomal RNA levels (Fig. 1a) and quantification of cellular RNAs (data not shown). In semi-quantitative (Fig. 1b) and quantitative (Fig. 1c, Supplementary Fig. S1) RT-PCR analyses, both a marked increase and decrease in the 3.5 kb RNA level were observed in PUF60-expressing cells at days 2 and 4 pt, respectively, compared to data obtained from northern blotting. No contamination of the transfected HBV plasmids in our RNA preparation was confirmed by no detection of amplified DNA without reverse transcription (Fig. 1b). At both time points, Sp1 RNA levels in PUF60-expressing cells were lower than those in control cells. Levels of HBV proteins such as HBs and HBc were also lower in the culture of PUF60-expressing cells (Supplementary Fig. S2). Immunoblotting showed that similar levels of PUF60, detectable as monomer and SDS-resistant dimer forms^11^, were expressed at days 2 and 4 pt in cells (Fig. 1b). Nuclear and cytoplasmic fractions of cells transfected with pUC-HB-Ce with or without pcDNA-F-PUF60 were isolated and 3.5 kb RNA levels in each fraction were determined. At day 1 pt, PUF60 expression resulted in a marked increase in the nuclear 3.5 kb RNA level (Fig. 1d, left). In contrast, at day 4 pt, PUF60 expression led to significantly (p < 0.01) low 3.5 kb RNA levels in both the nucleus and cytoplasm (Fig. 1d, right). Isolation of the nuclear and cytoplasmic fractions was confirmed by immunoblotting to detect each marker protein (Supplementary Fig. S3). A dose-dependent increase and decrease in the 3.5 kb RNA level by PUF60 expression from various concentrations of plasmids transfected were also observed (Fig. 1e). Impact of PUF60 on the 3.5 kb RNA expression was further assessed in other HBV genotypes (Fig. 1f). Increased 3.5 kb RNA levels at day 2 pt and subsequent decreased levels at day 4 pt in HBV-replicating cells with PUF60 expression were detected not only in HBV genotype C, but also HBV genotypes A and B.

**Figure 2:**
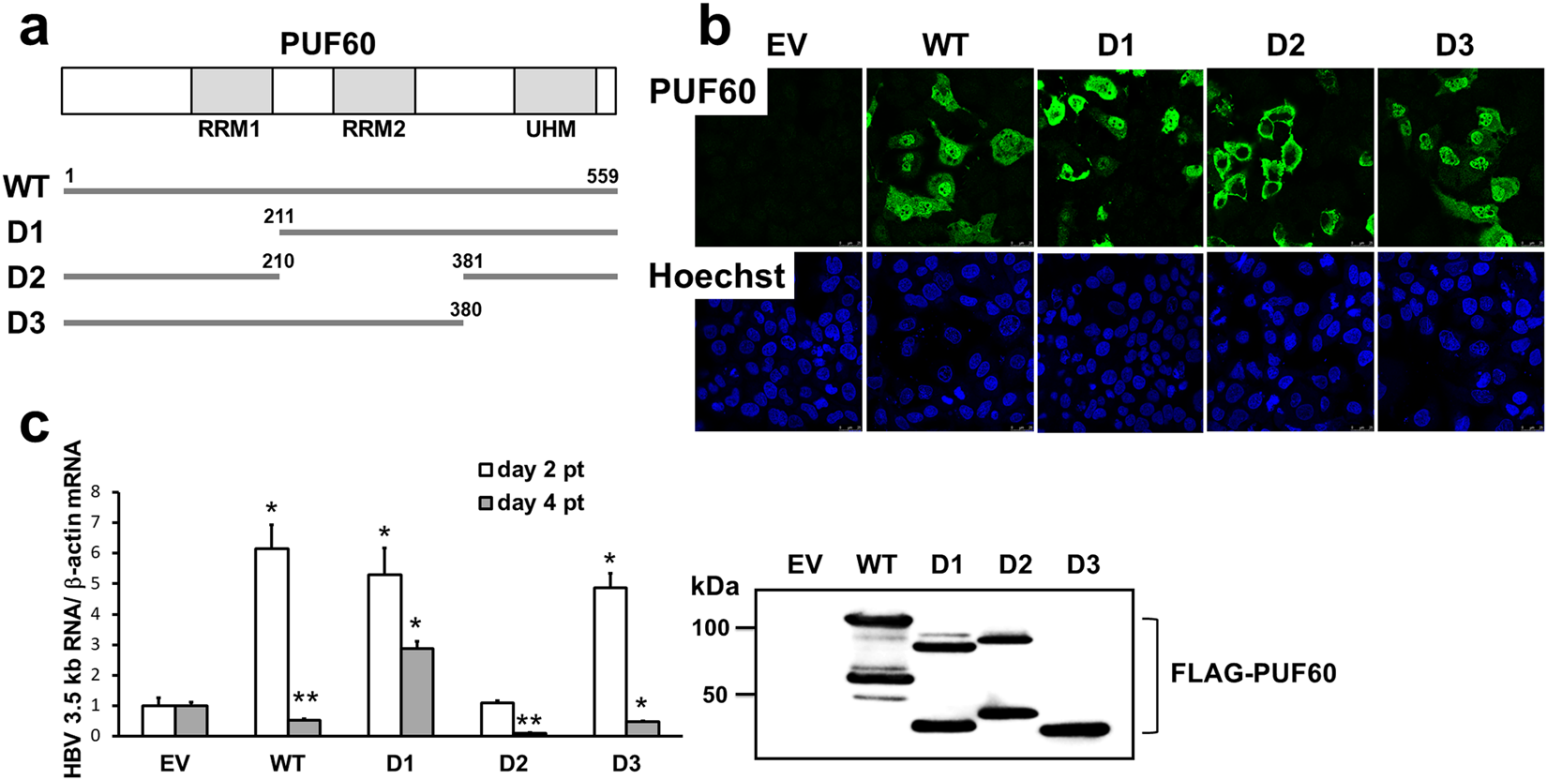
Effect of PUF60 deletion mutants on HBV 3.5 kb RNA expression. (**a**) Three PUF60 deletion mutants were used in this study. A schematic diagram of RNA recognition motifs (RRM1 and RRM2) and U2AF homology motif (UHM) within PUF60 is indicated at the top. (**b**) Subcellular localization of wild-type and mutant PUF60 strains was determined. At day 2 pt, cells were fixed and stained with Hoechst 33342, followed by immunostaining with anti-PUF60 antibody. (**c**) Effect of over-expression of PUF60 deletion mutants on 3.5 kb RNA expression at days 2 and 4 pt was evaluated in cells co-transfected with pUC-HB-Ce. Data are normalized to that of β-actin mRNA and the values in cells transfected with EV are set to 1. Values shown represent means ± SD obtained from three independent samples. Expression of each PUF60 deletion mutant was evaluated by immunoblotting. Full-length blot is presented in Supplementary Figure S16. Statistical significances compared with the control (EV) were shown. *p < 0.05, **p < 0.01, Student’s t test.

PUF60 is known as a member of the U2 small nuclear ribonucleoprotein auxiliary factor (U2AF) family and contains two canonical RNA recognition motifs (RRMs) at its N-terminal (aa 129–207) and central (aa 226–304) regions. An additional unusual RRM called the U2AF homology motif (UHM)^12^ is located at the C-terminus (aa 462–549) of PUF60. To identify the regions in PUF60 responsible for its effects on increased and decreased 3.5 kb RNA expression, PUF60 deletion mutants, pcDNA-F-PUF60-D1, -D2 and -D3, which encode PUF60 lacking one of the motifs (PUF60-D1, -D2 and -D3) indicated above, respectively, with a FLAG tag were constructed (Fig. 2a). The subcellular localization of wild-type and mutant PUF60 strains was determined by immunostaining with anti-PUF60 antibody (Fig. 2b). Wild-type PUF60 mainly localized to the nucleus and was partly present in the cytoplasm. The mutants PUF60-D1 and -D3 also mainly localized to the nucleus. In contrast, PUF60-D2 expression showed a homogeneous cytoplasmic distribution. It is thus likely that the second RRM, but not the first RRM and UHM within PUF60, is critical for its nuclear localization.

Effects of over-expression of each PUF60 deletion mutant on 3.5 kb RNA expression at days 2 and 4 pt were tested in cells co-transfected with pUC-HB-Ce (Fig. 2c). At day 2 pt, 3.5 kb RNA levels in cells expressing wild-type PUF60, PUF60-D1 or -D3 were 5- to 6-fold higher than that in control cells. In contrast, the 3.5 kb RNA level in cells expressing PUF60-D2 was comparable to that of control cells. At day 4 pt, although expression of wild-type PUF60, PUF60-D2 or -D3 led to decreases in the 3.5 kb RNA level, increased 3.5 kb RNA levels (e.g., 3-fold higher compared to that of the control) was maintained in cells expressing PUF60-D1. These results strongly suggest that the central (second) and N-terminal (first) RRMs, respectively, are important for up-regulation of 3.5 kb RNA expression at early time points after introduction of the HBV genome (day 2 pt) and its subsequently decreased effect on 3.5 kb RNA observed at a later time point (day 4 pt).

**Figure 3:**
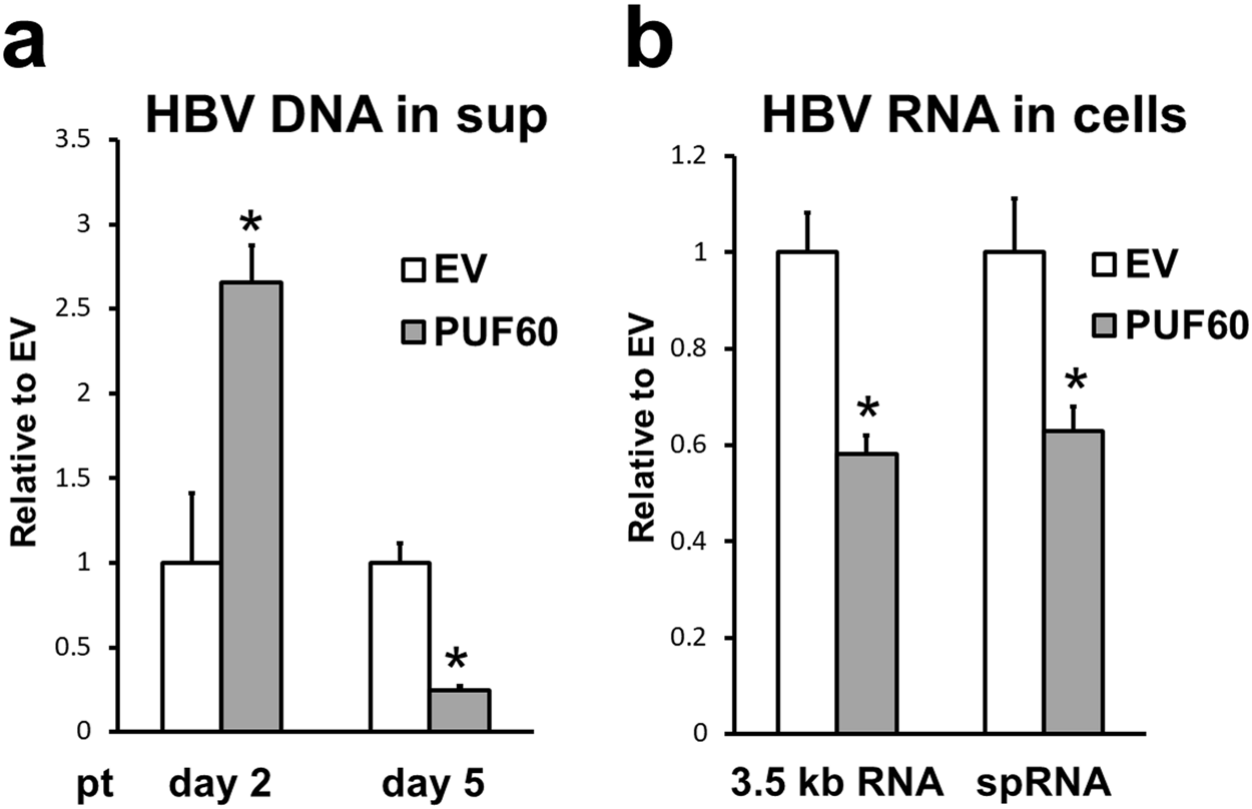
Effect of PUF60 on HBV production. (**a**) Particle-associated HBV DNA in culture supernatants of cells transfected with pUC-HB-Ce and pcDNA-F-PUF60 or empty vector (EV) was quantitatively measured at days 2 and 5 pt. (**b**) After 12 h of transfection with pcDNA-F-PUF60 or EV, HepG2-hNTCP-C4 cells were infected with HBV and cultured for 5 days, and total cellular RNA was analyzed by RT-qPCR to determine levels of 3.5 kb RNA (left) and spRNAs (right). (**a**) and (**b**) Values shown represent means ± SD obtained from three independent samples. Statistical differences compared with the control (EV) are shown. *p < 0.05, Student’s t test.

Next, to determine the effect of PUF60 on HBV production, particle-associated HBV DNA in culture supernatants of cells transfected with pUC-HB-Ce with or without pcDNA-F-PUF60 was quantitatively measured (Fig. 3a). The results were comparable to those of 3.5 kb RNA in cells (Fig. 1a–c). Although the viral DNA level at day 2 pt was 2.5-fold higher in the culture with PUF60 expression compared to that of the control culture, the DNA level in the supernatant of PUF60-expressing cells at day 5 pt was 5-fold lower than that of control cells. Collectively, these findings suggest involvement of PUF60 in both positive and negative regulation of HBV replication.

We next used the HBV infection system with NTCP-expressing HepG2 cells, HepG2-hNTCP-C4 cells^13^, to assess the influence of PUF60 on viral infection (Fig. 3b). After 12 h of transfection with pcDNA-F-PUF60 or an empty vector, HepG2-hNTCP-C4 cells were inoculated with HBV prepared from the culture supernatant of HepG38.7-Tet^14^ and cultured for 5 days. Total cellular RNA levels were then determined by reverse-transcription quantitative PCR (RT-qPCR). As expected, both 3.5 kb RNA and Sp1 RNA levels in infected cells with PUF60 expression were significantly lower than those in control infected cells. At earlier time points, such as day 2 post-infection, it was difficult to assess the influence of PUF60 expression since basal levels of HBV RNAs were quite low in this setting.

### PUF60 as a positive regulator of HBV core promoter activity

**Figure 4:**
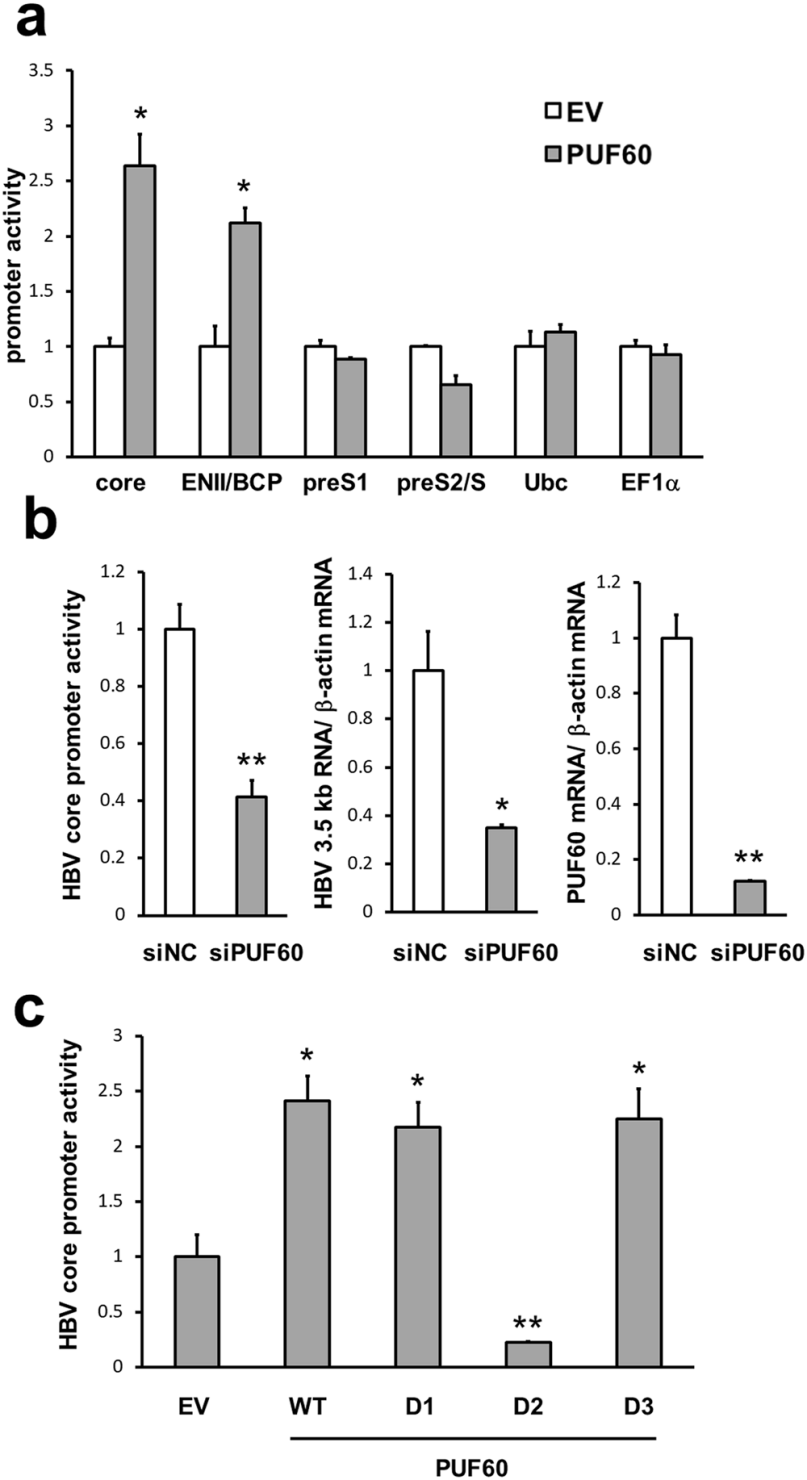
Up-regulation of HBV core promoter activity induced by PUF60. (**a**) Effect of PUF60 expression on HBV or cellular promoter activities was analyzed by transfection of HuH-7 cells with the luciferase reporter carrying the entire core promoter (nt 900–1817), ENII/BCP (nt 1627–1817), preS1 promoter (nt 2707–2847), preS2/S promoter (nt 2937–3204), human ubiquitin C promoter or human elongation factor 1α promoter and pcDNA-F-PUF60 or empty vector (EV). Reporter activities in the cells were measured at 24 h pt. Values are normalized to total protein concentrations in cell lysates. (**b**) Knockdown effect of PUF60 on core promoter activity (left) and 3.5 kb RNA expression (middle) as well as knockdown efficiency of PUF60 (right) were assessed. At 2 days after introducing PUF60 siRNAs (siPUF60) or its negative control (siNC), HuH-7 cells were transfected with pGLHBp900/1817 or pUC-HB-Ce and then reporter activities and RNA levels, respectively, were measured after 2 days of further culture. PUF60 mRNA expression was also determined. (**c**) Effect of PUF60 deletion on activation of the core promoter was assessed. HuH-7 cells were transfected with pGLHBp900/1817 and a plasmid expressing either wild-type PUF60, PUF60-D1, -D2, -D3 or EV. Reporter activities were measured at day 1 pt. (**a**)–(**c**) Data are normalized to that of β-actin mRNA and the values in cells transfected with EV or siNC are set to 1. All assays were performed in triplicate and results are presented as means ± SD. Statistical differences compared with the control (EV or siNC) are shown. *p < 0.05, **p < 0.01, Student’s t test.

Based on the positive effect of PUF60 on 3.5 kb RNA, in particular at the nuclear level, at the early phase pt (Fig. 1a–d), we next investigated whether PUF60 plays a role in transcriptional regulation of 3.5 kb RNA. Effect of PUF60 on HBV promoter activities was analyzed by transfection of HuH-7 cells with a luciferase reporter carrying either the entire core promoter (nt 900–1817), ENII/basal core promoter (BCP) (nt 1627–1817), preS1 promoter (nt 2707–2847) or preS2/S promoter (nt 2937–3204) with or without pcDNA-F-PUF60. Reporter activities in the cells were measured at 24 h pt (Fig. 4a). The activities of both the entire core promoter and ENII/BCP were significantly higher in cells over-expressing PUF60. In contrast, preS1 and preS2/S promoter activities were not affected by PUF60 expression. PUF60 also had little or no influence on human ubiquitin C promoter and human elongation factor 1α promoter activities. At day 4 pt, no significant effect of PUF60 expression on core promoter activity was observed (Supplementary Fig. S4). Effects of PUF60 knockdown on core promoter activity and 3.5 kb RNA expression were further assessed (Fig. 4b). As expected, at day 2 pt, siRNA-based silencing of PUF60 reduced both promoter activity and the 3.5 kb RNA level. In contrast, at day 4 pt, PUF60 knockdown led to a marginal effect on the core promoter activity but, somewhat unexpectedly, reduced the 3.5 kb RNA level (Supplementary Fig. S5). Knockdown efficiency of PUF60 gene was confirmed by immunoblotting (Supplementary Fig. S6).

PUF60 deletion experiments (Fig. 2) identified protein regions critical for positive and negative regulation of 3.5 kb RNA expression by PUF60. Effect of PUF60 deletions on activation of the core promoter was further assessed (Fig. 4c). Consistent with the result shown in Fig. 2c, the increase in core promoter activity induced by PUF60 expression was cancelled with expression of PUF60-D2. Thus, it appears that up-regulation of core promoter activity mediated by the central RRM within PUF60 led to an increase in the 3.5 kb RNA level seen at day 2 pt with the PUF60-expressing plasmid.

### Involvement of TCF7L2 in up-regulation of core promoter activity potentially via interaction with the ENII region and PUF60

**Figure 5:**
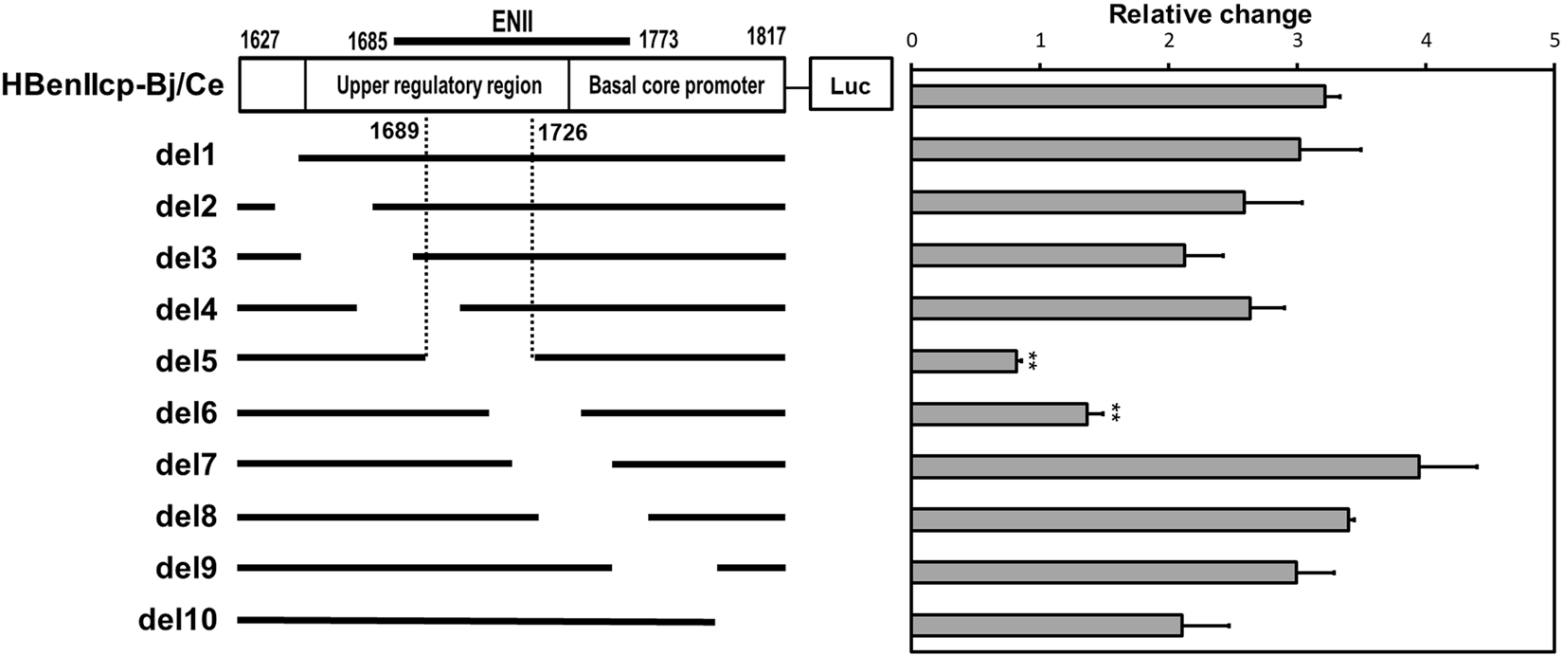
Identification of the ENII/BCP element(s) responsible for transcriptional regulation by PUF60. A series of reporter constructs with partial deletions within ENII/BCP were generated (left). Luciferase activities were determined in cells transfected with each reporter construct and pcDNA-F-PUF60 or empty vector (EV) at day 2 pt (right). Relative changes in reporter activities induced by PUF60 calculated as the ratios of reporter activities in cells expressing PUF60 to those in control cells are shown.

To address the molecular mechanism underlying PUF60 regulation of the core promoter, a series of reporter constructs with partial ENII/BCP deletions were first generated (Fig. 5, left) to identify the element(s) responsible within the ENII/BCP sequence for transcriptional regulation by PUF60. Luciferase activities were determined by co-transfection of HuH-7 cells with or without pcDNA-F-PUF60 (Fig. 5, right). Although most deletions tested maintained the increase in reporter activity by PUF60 expression, HBenIIcp-del-5 (deletion of nt 1689–1726) and −6 (deletion of nt 1710–1742) cancelled the effect by PUF60. This result indicates that the nt 1689–1742 region, located in ENII, is important for transcriptional regulation of 3.5 kb RNA mediated by PUF60.

**Figure 6:**
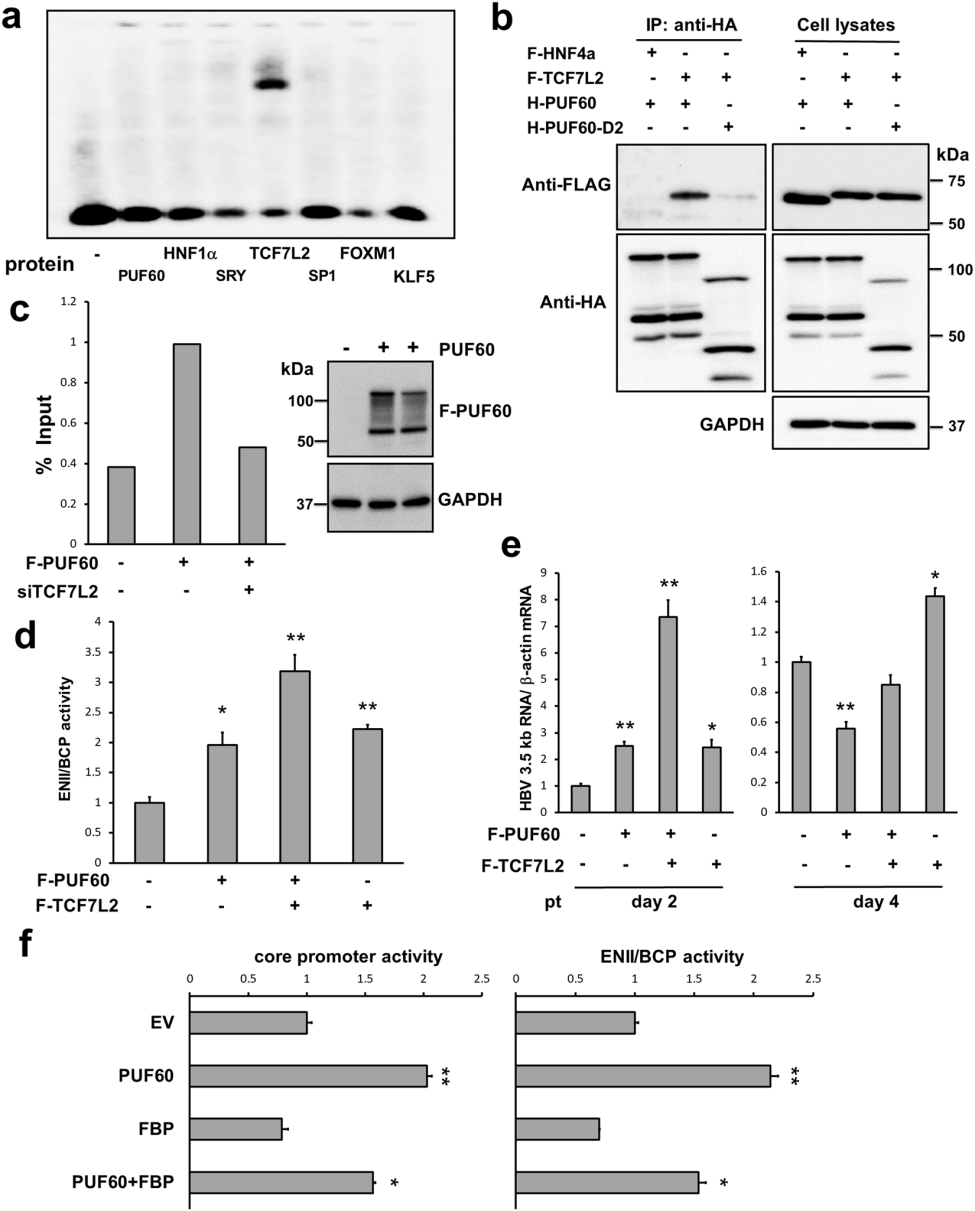
Involvement of TCF7L2 in up-regulation of core promoter activity. (**a**) Transcription-related proteins from database search (HNF1α, SRY, TCF7L2, SP1, FOXM1 and KLF5) and PUF60 were synthesized *in vitro* and used for the gel shift assay with an end-labeled oligonucleotide probe (nt 1689–1726). (**b**) Interaction of PUF60 with TCF7L2 in cells was tested. Cells transfected with pcDNA-HA-PUF60 or -PUF60-D2 and pcDNA-F-TCF7L2 or -HNF4α plasmids were lysed at day 2 pt and subjected to immunoprecipitation (IP) with anti-HA antibody. Resulting precipitates and whole cell lysates were examined by immunoblotting using anti-FLAG, anti-HA or anti-GAPDH antibody. (**c**) ChIP assay was performed to determine recruitment of PUF60 to the core promoter in cells. After 2 days with or without knockdown of TCF7L2, cells were transfected with pcDNA-F-PUF60 or empty vector (EV). Further 2 days later, cell lysates were immunoprecipitated with anti-FLAG antibody, and HBV DNA in the precipitates was measured by qPCR. FLAG-PUF60 and GAPDH in the precipitates were detected by immunoblotting. (**d**) Effect of TCF7L2 expression on ENII/BCP activity was determined by the reporter assay. Cells were transfected with pcDNA-F-PUF60 or -TCF7L2 or both and pGLHBp1627/1817. At 24 h pt, *Renilla* luciferase activities in cells were measured. (**e**) Effect of TCF7L2 over-expression with or without PUF60 on 3.5 kb RNA expression was determined. Cells were transfected with pcDNA-F-PUF60 or -TCF7L2 or both and pUC-HB-Ce. At day 2 or 4 pt, total RNA was extracted and HBV 3.5 kb RNA level was assessed by RT-qPCR. Results were normalized to that of β-actin mRNA. (**f**) Effect of FBP over-expression with or without PUF60 on core promoter and ENII/BCP activities was assessed. Cells were transfected with pcDNA-HA-FBP or -F-PUF60 or both and pGLHBp900/1817 or pGLHBp1627/1817. Luciferase activities in cell lysates were measured at day 2 pt. (**d**)–(**f**) The values in cells transfected with EV are set to 1. Results are presented as means ± SD from at least three independent samples. Statistical differences compared with the negative control (EV only) are shown. *p < 0.05, **p < 0.01, Student’s t test. Full-length blots in (**b**) and (**c**) are presented in Supplementary Figures S17 and S18, respectively.

From our transcription factor database search, six transcription-related proteins, HNF1α, SRY, TCF7L2, SP1, FOXM1 and KLF5, were predicted to be possible binding factors within the nt 1689–1742 region of ENII. Thus, whether these proteins and PUF60 are able to bind directly to the sequence was assessed by the gel shift assay using *in vitro* synthesized proteins and end-labeled oligonucleotide probes spanning the nt 1689–1726 and nt 1710–1742 regions. Among the proteins tested, TCF7L2^15^, a member of the TCF family of transcription factors that is predicted to bind to nt 1708–1713 (TTCAAAG) from the search program, was found to bind to the nt 1689–1726 sequence (Fig. 6a) but not to the nt 1710–1742 sequence (data not shown). Other proteins including PUF60 did not bind to these sequences. From these results, we hypothesized that PUF60 possibly accesses ENII of the HBV core promoter via interaction with ENII-binding partner, TCF7L2, leading to up-regulation of 3.5 kb RNA transcription.

To address this hypothesis, interaction between PUF60 and TCF7L2 in cells was tested (Fig. 6b). PUF60 co-precipitated with TCF7L2 but not with HNF4α. PUF60-TCF7L2 interaction was largely cancelled in PUF60-D2-expressing cells (Fig. 2a), which cannot increase the 3.5 kb RNA level (Fig. 2c) or core promoter activity (Fig. 4c). The Chromatin immunoprecipitation (ChIP) assay with or without knockdown of TCF7L2 was further performed to determine PUF60 recruitment to the ENII region (Fig. 6c). Amplified DNA covering the nt 1589–1828 region was detectable after immunoprecipitation of cell lysates with or without expressing FLAG-tagged PUF60 with an anti-FLAG antibody. Additionally, the DNA level was clearly lower in TCF7L2-knockdown cells, indicating involvement of TCF7L2 in PUF60 recruitment to the ENII region, which is important for 3.5 kb RNA expression.

Involvement of TCF7L2 in ENII/BCP activity was examined by the reporter assay in which luciferase activities of the cells were measured at 24 h pt (Fig. 6d). Over-expression of either TCF7L2 or PUF60 led to a comparable increase in ENII/BCP activity, which was further increased by co-expression of both TCF7L2 and PUF60. Effects of TCF7L2 over-expression with or without PUF60 on 3.5 kb RNA expression at days 2 and 4 pt were assessed in cells co-transfected with pUC-HB-Ce (Fig. 6e). As expected, at day 2 pt, the 3.5 kb RNA level in cells co-expressing both TCF7L2 and PUF60 were markedly higher than those in cells expressing either TCF7L2 or PUF60, in which significantly increased 3.5 kb RNA levels were observed compared to control cells. At day 4 pt, in contrast to the effect of PUF60 shown in Fig. 1c, the increase in 3.5 kb RNA levels induced by TCF7L2 expression alone was maintained.

These results strongly suggest that PUF60 acts as a positive regulator of ENII/BCP activity during 3.5 kb RNA transcription cooperatively with TCF7L2. It has been shown that PUF60, also known as FIR, plays a role in c-myc transcription via interaction with FBP, which targets FUSE located upstream of the c-myc promoter^10^. Although no typical FUSE-like sequence was detected within the HBV genome, we further examined whether FBP is involved in 3.5 kb RNA expression (Fig. 6f). No significant impact on core promoter and ENII/BCP activities by over-expression of FBP was found. A proper expression of HA-tagged FBP from the expression plasmid used was confirmed (Supplementary Fig. S7). The results indicated that FBP does not participate in the PUF60-dependent mechanism on ENII/BCP regulation.

### Role of PUF60 on HBV 3.5 kb RNA degradation

**Figure 7:**
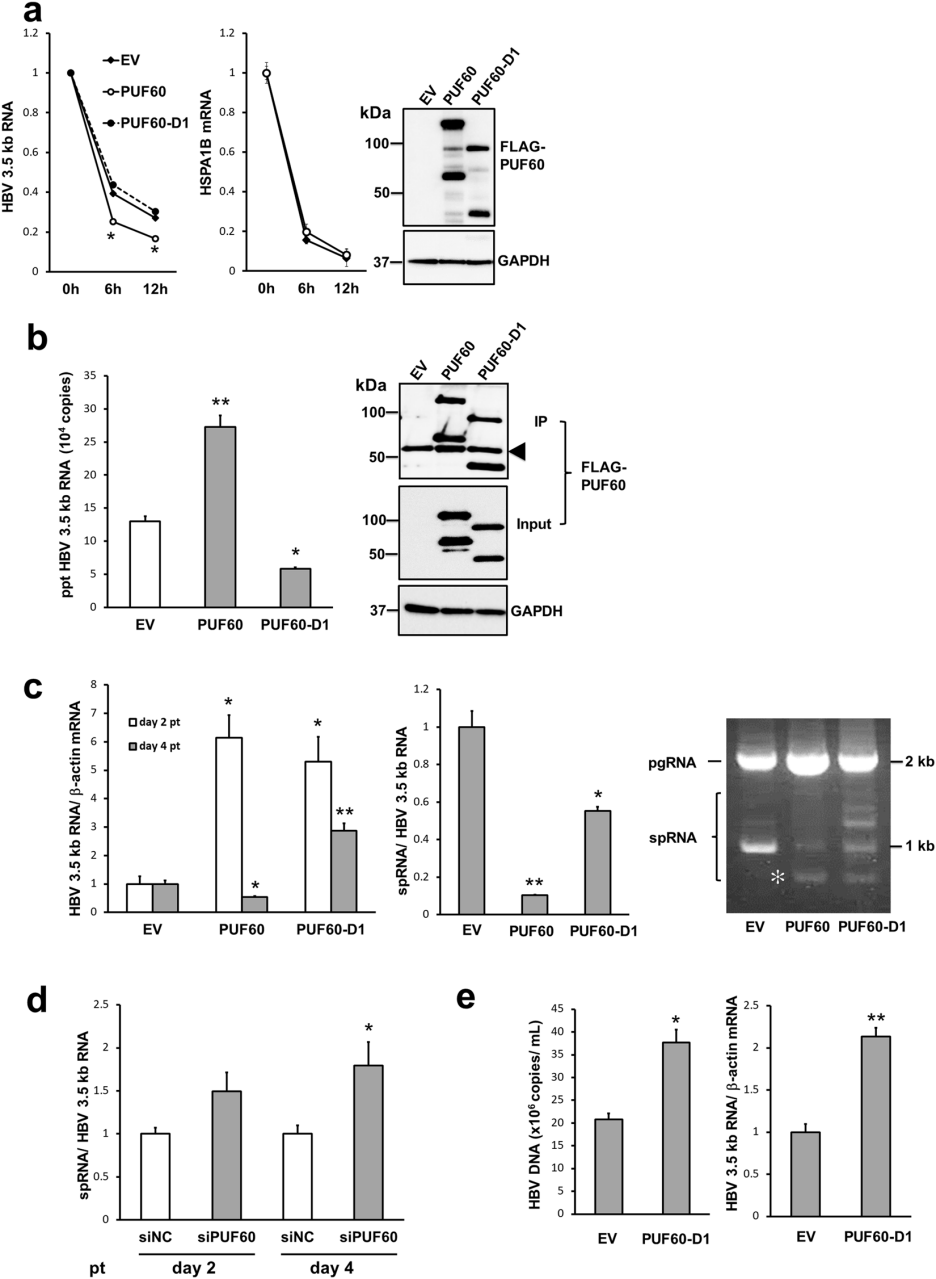
Involvement of PUF60 in HBV 3.5 kb RNA degradation. (**a**) Time course changes in HBV 3.5 kb RNA and HSPA1B mRNA levels were determined. At day 2 pt with pUC-HB-Ce and pcDNA-F-PUF60, -PUF60-D1 or empty vector (EV), aliquots of cells were harvested (0 h) and the remaining cells were treated with actinomycin D, followed by 6- or 12 h culture. At each time point, total RNA was extracted and the RNA levels were assessed by RT-qPCR. The values of each group at 0 h were set to 1. Expression of PUF60, PUF60-D1 and GAPDH was detected by immunoblotting. (**b**) Interaction of PUF60 with 3.5 kb RNA in cells was examined. HuH-7 cells were transfected with pUC-HB-Ce and pcDNA-F-PUF60, -PUF60-D1 or EV, followed by immunoprecipitation (IP) with anti-FLAG antibody at day 2 pt. HBV 3.5 kb RNA level in the precipitates was determined by RT-qPCR (left). The precipitates and whole cell lysates used in IP were examined by immunoblotting using anti-FLAG or anti-GAPDH antibody. An arrowhead indicates non-specific bands. (**c**) Effects of PUF60 and PUF60-D1 on 3.5 kb RNA and spRNA levels were tested. Cells were transfected with pUC-HB-Ce and pcDNA-F-PUF60, -PUF60-D1 or EV, followed by RT-qPCR for 3.5 kb RNA at days 2 and 4 pt (left). At day 2 pt, spRNA levels in each transfectant were determined by RT-qPCR (middle) and semi-quantitative RT-PCR (right). The values in cells transfected with EV are set to 1. (**d**) Effect of PUF60 knockdown on the ratio of spliced/unspliced 3.5 kb RNA at day 2 and 4 pt. (**e**) Effect of PUF60-D1 over-expression on HBV production was assessed. Particle-associated HBV DNA in culture supernatants as well as 3.5 kb RNA in these cells transfected with pUC-HB-Ce and pcDNA-F-PUF60-D1 or EV were measured at day 5 pt. (**a**)–(**e**) Assays were performed in triplicate and results are presented as means ± SD. Statistical differences compared with the control (EV) are shown. *p < 0.05, **p < 0.01, one-way ANOVA followed by Tukey’s test (**a**) or Student’s t test (**b**–**e**). Full-length blots in (**a**), (**b**) and (**c**) are presented in Supplementary Figures S19, S20 and S21.

In addition to the positive effect on 3.5 kb RNA expression, the findings described above demonstrate that PUF60 potentially has a negative role on the steady state level of 3.5 kb RNA during the HBV life cycle. To address the mechanism underlying this negative regulation, the effect of PUF60 expression on 3.5 kb RNA decay or degradation was determined (Fig. 7a). Cells replicating the HBV genome with or without PUF60 expression were treated with actinomycin D to arrest de novo RNA synthesis at day 2 pt, followed by RNA isolation at 0, 6 and 12 h after addition of actinomycin D. PUF60 expression resulted in faster degradation of 3.5 kb RNA (Fig. 7a, left) but not of cellular mRNA of constitutively expressed heat shock protein family A member 1B (HSPA1B) (Fig. 7a, right). At day 4 pt, it appeared difficult to evaluate effect of PUF60 expression on decay of the 3.5 kb RNA since the RNA level in PUF60-expressing cells was quite low even at 0 h (Supplementary Fig. S8). To further determine the effect of PUF60 expression on decay of HBV RNA, *in vitro* synthesized HBV RNA was electroporated into cells after 4 days of the culture that was transfected either with pcDNA-F-PUF60 or an empty vector, followed by monitoring the viral RNA level until 20 h after the RNA electroporation. As shown in Supplementary Fig. S9, the decay of HBV RNA in PUF60-expressing cells was faster compared to that in the control cells. As indicated in Fig. 2, the N-terminal RRM region within PUF60 is important for its inhibitory effect on 3.5 kb RNA expression. The effect on 3.5 kb RNA degradation was cancelled by deleting the RRM region (PUF60-D1; Fig. 7a, left). Interaction of PUF60 with 3.5 kb RNA was detectable in HBV genome-replicating cells that expressed the full-length PUF60 but not PUF60-D1 by immunoprecipitation and RT-qPCR analyses (Fig. 7b). Time course changes in the 3.5 kb RNA level in cells replicating the viral genome in the presence of PUF60-D1 was compared with that in the presence of full-length PUF60 (Fig. 7c, left). At day 2 pt, the 3.5 kb RNA level in cells expressing PUF60-D1 was comparable to that in cells expressing full-length PUF60 and markedly higher than control cells without PUF60 expression. Interestingly, at day 4 pt, in contrast to the decreased level of 3.5 kb RNA in cells expressing full-length PUF60 compared to control cells, PUF60-D1 expression maintained the increased level of 3.5 kb RNA seen at day 2 pt. These findings demonstrate a critical role of the N-terminal RRM region within PUF60 in HBV 3.5 kb RNA degradation.

Ratio of spliced RNAs/unspliced 3.5 kb RNA, was also compared in cells expressing full-length PUF60 versus PUF60-D1 (Fig. 7c, middle). The ratio in cells expressing PUF60-D1 was significantly higher (p < 0.05) than that of cells expressing full-length PUF60 but was still lower than that of the control. By semi-quantitative RT-PCR, not only a change in the ratio of spliced/unspliced 3.5 kb RNA but variation of alternative splicing induced by PUF60- or PUF60-D1 expression were observed (Fig. 7c, right, Supplementary Fig. S10). By cDNA cloning and sequencing, the alternative splicing induced by PUF60 expression (an asterisk in Fig. 7c, right) was found to contain an intron with 0.2 kb longer than that in Sp1 RNA (Supplementary Fig. S11). Its splice site did not conform to consensus splicing dinucleotides and a homologous sequence CACC was found around its junction site at nt 2107–2110 and 336–339. To our knowledge, it appears that this RNA processing has not been reported in HBV-replicating cells as well as in liver tissues of hepatitis B patients to date. Expression of PUF60 or PUF60-D1 resulted in decreasing Sp1 RNA, which was dominant in the control cells, but in increasing other spliced RNAs, indicating involvement of PUF60 on splicing events of HBV pregenomic RNA. In contrast, PUF60 knockdown resulted in increase of the spliced/unspliced 3.5 kb RNA ratio (Fig. 7d, Supplementary Fig. S12). These findings suggest the N-terminal RRM region is important for regulation of 3.5 kb RNA splicing mediated by PUF60 but additional region(s) in PUF60 may also be involved in this regulation. It may be likely that PUF60-D1 expression contributes to an increased level of HBV production. To address this issue, we evaluated HuH-7 cells replicating the HBV genome transfected with the PUF60-D1 expression plasmid. Approximately 2-fold higher levels of particle-associated HBV DNA in the culture supernatant as well as 3.5 kb RNA in these cells compared to cells without PUF60-D1 expression was observed at day 5 pt (Fig. 7e).

Collectively, these results suggest that human hepatoma cells with ectopic expression of cell-derived proteins such as PUF60-D1, which contribute to up-regulation of 3.5 kb RNA but have no effect on its degradation, are potentially useful to increase HBV production.

## Discussion

In general, nucleic acid binding proteins play roles in a variety of cellular processes, including transcriptional regulation, pre-mRNA splicing and nucleic acid transport. Although proteins that bind both mRNAs and their encoding promoters are considered to have functional advantages or flexibility in generating cellular responses, DNA- and RNA-binding proteins have been largely studied independently in modulating gene expression. In the course of study to determine roles of host proteins that have DNA- and RNA-binding properties in HBV replication, we found that PUF60 potentially functions as a versatile regulator of both transcriptional and post-transcriptional steps of HBV pregenome expression.

In this study, we demonstrated that PUF60 expression leads to: 1) up-regulation of core promoter activity, 2) suppression of pregenome-derived RNA splicing and 3) promotion of pregenome degradation. When the 1.24-fold HBV genome plasmid was introduced into cells with co-transfection of the PUF60-expression vector, the 3.5 kb RNA level increased at an early time point, such as days 1–2 pt, but subsequently decreased compared to control HBV-replicating cells. To our knowledge, this is the first study to demonstrate involvement of a host factor in not only positively regulating gene expression and replication of virus, but also the negative regulation of the same viral life cycle. While why both decreased and increased expression of PUF60 lowered the 3.5 kb RNA at day 4 pt (Fig. 1, Supplementary Fig. S5) is unclear to date, critical contributions of PUF60 to diverse biological pathways in transcriptional- and post-transcriptional processes during the viral replication potentially in a time-dependent manner might lead to such an unusual regulatory paradigm.

PUF60 is a splicing factor that associates with splicing factors involved in early spliceosome assembly and plays a role in the recognition of the 3′ splice site during recruitment of small nuclear ribonucleoproteins (snRNPs) to the intron for splicing^16,17^. PUF60 is classified as a member of the U2AF protein family, where canonical RRMs with distinct features of protein recognition are conserved^12^. U2AF-related proteins are potentially involved in changes in available splice sites by preventing initial binding of U1 snRNP and U2AF during spliceosome assembly.

Although mRNA turnover is critical for gene expression in eukaryotic cells, contribution of decay factors to mRNA degradation machineries remains poorly understood because of their complexity. In particular, evidence for roles of U2AF-related proteins in mRNA degradation is quite limited. T-cell intracellular antigen 1 (TIA1), known to possess U2AF homology motifs, has been shown to contribute to modulation of the mRNA level of programmed cell death 4 (PDCD4) through binding to PDCD4 3′ UTR mRNA^18^. Competition between TIA1 and another RNA-binding factor, HuR, for binding on PDCD4 mRNA is thought to be important for fine-tuning PDCD4 expression in cells. Additionally, TIA1 has been shown to contribute to HBs expression possibly via interaction with a particular HBV RNA sequence, post-transcriptional regulatory element (PRE)^19^.

RNA decay mechanisms such as innate immune recognition, nonsense-mediated decay, RNA exosome and canonical RNA decay machinery are now recognized to play an important role in antiviral defense in mammalian cells. For example, as anti-HBV defense mechanisms, the zinc finger antiviral protein ZAP has been shown to target HBV 3.5 kb RNA, resulting in RNA decay^20^, as seen in retroviruses, alphaviruses and filoviruses^21–23^. Cytidine deaminase possibly triggers HBV RNA degradation by tethering the RNA exosome to the viral protein/RNA complex^24^. Non-stop-mediated RNA quality control is potentially involved in degradation of the viral X mRNA at the RNA exosome complex^25^. To our knowledge, this study is the first to reveal the role of PUF60 in mRNA degradation, and PUF60-mediated degradation of viral RNAs might be a novel type of antiviral defense mechanism. PUF60 was first identified as a 60-kDa protein that efficiently binds to the poly-U tract. However, no typical or consensus motif for PUF60 binding is observed in HBV RNAs. To determine the HBV RNA degradation mechanism mediated by PUF60, we found direct binding of PUF60 to PRE within HBV RNA but not with its reverse sequence in the *in vitro* assay (Supplementary Fig. S13). Although HBV PRE has been reported to be involved in viral mRNA regulation such as nuclear export, mRNA stability and splicing^26–29^, little is understood about the underlying molecular mechanisms. Further study to elucidate the significance of PUF60-PRE interaction on stability of viral RNAs and PUF60-dependent pathway of RNA degradation is currently underway.

In addition to roles as an RNA-binding factor, it has been shown that PUF60 potentially controls the expression of c-myc at the transcriptional step by inhibiting the transcription factor, FBP^10^. PUF60 is thus termed FIR. It is thought that the interplay between FUSE, FBP and FIR/PUF60 influences the timing and level of c-myc expression^30^.

Our findings suggest that PUF60 positively regulates ENII/BCP activity via interaction with TCF7L2, which can bind directly to the ENII sequence. Interaction of PUF60 with TCF7L2 was cancelled by deleting the aa 210–281 region of PUF60 (Fig. 5), which is essential for the positive regulation of 3.5 kb RNA expression (Fig. 2c). Despite the lack of direct binding between PUF60 and the ENII sequence (Fig. 6a), results of the ChIP assay indicated that PUF60 can be recruited to the ENII region of the HBV genome, and this recruitment is impaired by knockdown of TCF7L2 (Fig. 6c). The highest positive impact on ENII/BCP activity and 3.5 kb RNA expression was found in cells co-expressing PUF60 and TCF7L2 compared to cells over-expressing either PUF60 or TCF7L2 alone (Fig. 6d and e). It is also noted that the consensus DNA sequence for TCF7L2 binding is well conserved within the ENII region among HBV isolates including HBV genotypes A to D.

TCF7L2 is a key member of the TCF family of transcription factors, which are known as downstream transcriptional effectors of Wnt signaling^31^ and have been shown to bind DNA directly and recruit multiple transcriptional factors such as GATA3, β-catenin, HNF4α and FOXO1^32–34^. Genetic variants of TCF7L2 showed the strongest association with type 2 diabetes/gestational diabetes mellitus to date. Several studies have demonstrated that, in the liver, TCF7L2 potentially serves as an important regulator of glucose homeostasis by regulating proinsulin production and processing^35,36^. It is further suggested that TCF7L2 also plays metabolic roles in lipid and amino acid metabolism, and such diverse roles are possibly accomplished via its interactions with various transcriptional factors as shown above. Here, we found that TCF7L2 also plays a role in viral transcription. TCF7L2 functions as a positive regulator of ENII/BCP activity in HBV via binding to the ENII region. Moreover, its interaction with PUF60 leads to further acceleration of ENII/BCP activity and 3.5 kb RNA expression. It will be of interest to determine if 3.5 kb RNA expression regulated by TCF7L2 has an influence on metabolic gene expression mediated by TCF7L2 and if the interaction with PUF60 is also involved in TCF7L2-dependent regulation of cellular gene expression. Given the role as a host restriction factor that limits HBV replication, the positive regulation of ENII/BCP activity induced by PUF60, coupled with TCF7L2, might be an evolutionally acquired strategy to avoid or reduce antiviral effects via the RNA decay pathway.

In conclusion, we identified PUF60 as a versatile regulator of the HBV life cycle, capable not only of transcriptional up-regulation of 3.5 kb RNA expression, but also post-transcriptional involvement including accelerating 3.5 kb RNA decay and suppressing 3.5 kb RNA splicing. It appears that PUF60 potentially changes the balance of the viral promoter activity and RNA decay in a time-dependent manner. Although further detailed analyses to understand the regulatory mechanisms of HBV life cycle mediated by PUF60 are required, these findings lead to insight on the functional linkage between transcriptional and post-transcriptional regulations on viral replication and a potential mechanism(s) to control antiviral host defense and viral persistence.

## Methods

### Plasmids

Plasmids containing the 1.24-fold HBV genomes derived from HBV genotypes Ae and Bj, pUC-HB-Ae and pUC-HB-Bj^37,38^, respectively, were gifts from Dr. Mizokami (National Center for Global Health and Medicine, Japan). pUC-HB-Ce, which contains the 1.24-fold HBV genome derived from a consensus sequence of HBV genotype Ce, was designed in accordance with the most common nucleotide observed among HBV genotype Ce clones (AB014381, AB205124, AB033551, AB198081, AY596108, AB198080, AB222714 and AY066028) at each position and was artificially synthesized by Eurofins Genomics (Ebersberg, Germany). DNA fragments of HBV core promoter derived from HBV genotypes B and C were designed in accordance with the most common nucleotide among HBV genotypes Bj and Ce, respectively, via searching the database of HBV sequences, and were then synthesized by Eurofins Genomics. To construct pGL4.74-HBpg-Ce, synthesized fragments corresponding to the nt 900–1817 region of the HBV genome digested by KpnI and HindIII were inserted upstream of the luciferase reporter gene of pGL4.74 (Promega, Madison, WI, USA). To construct pGL4.74-HBenIIcp-Bj/Ce, synthesized fragments corresponding to the nt 1627–1817 region were amplified by PCR and cloned into pGL4.74 as described above. The sequences of preS1 and preS2/S promoters were obtained from nt 2707–2847 and nt 2937–3204 regions, respectively, from a consensus sequence of genotype Ce. Promoter sequences of human ubiquitin C and human elongation factor 1α subunit were obtained from pUB6 and pEF6 (Thermo Fisher Scientific, Waltham, MA, USA), respectively, and subsequently inserted into pGL4.74. A series of deletion mutants, pGL4.74-HBenIIcp-Bj-D1–D10 (Fig. 5a), were generated based on pGL4.74-HBenIIcp-Bj. To create the PUF60 expression plasmid pcDNA-F-PUF60, the cDNA sequence of human PUF60 (Gene ID: 22827) was amplified by PCR using HuH-7 cells as the template, followed by digestion with HindIII and XbaI and subsequent insertion into pcDNA3.1 (Thermo Fisher Scientific). Plasmids expressing PUF60 deletion mutants were generated via several PCRs using pcDNA-F-PUF60 as the template, resulting in pcDNA-F-PUF60-D1, -D2 and -D3 (Fig. 2a). To create the FBP expression plasmid pcDNA-HA-FBP, the cDNA sequence of human FBP (Gene ID: 8880) was amplified by PCR as described above, followed by digestion with HindIII and XbaI and subsequent insertion into pcDNA3.1. pcDNA-F-TCF7L2 is kindly gift from Prof. Peggy Farnham (University of Southern California). Expression plasmid for HNF4α was generated previously^39^.

### Cell culture, transfection and RNA interference

Human hepatoma derived cells [HuH-7, HepG2, HepG38.7-Tet and HepG-hNTCP-C4^13^] were maintained in Dulbecco’s modified Eagle medium (DMEM) supplemented with 10% fetal bovine serum. Cells (1 × 10^5^ cells/well in a 24-well plate) were transiently transfected with 1 µg of plasmid DNA mixed with Lipofectamine LTX (Thermo Fisher Scientific). Synthetic siRNAs were provided by Ambion (Thermo Fisher Scientific) and were transfected into cells using Lipofectamine RNAiMAX Reagent (Thermo Fisher Scientific).

### HBV infection

The culture supernatant of HepG38.7-Tet cells^14^ was concentrated using Amicon Ultra-15 Centrifugal Filter Devices (MILLIPORE, Darmstadt, Germany) and the resulting HBV sample (HBV DNA copies 2 × 10^8^/ml) was used as an inoculum for infection assays. HepG2-hNTCP-C4 cells cultured in a 24-well collagen-coated plate were transfected with pcDNA-F-PUF60 and then inoculated with the HBV sample (50 µl) in DMEM containing 4% polyethylene glycol (PEG) 8000 (Promega) after 12 h of transfection. The cells were washed 3 times with PBS after 24 h of infection and then subjected to RT-qPCR after 96 h of further culture.

### Quantification of HBV DNA and RNA

Quantification of HBV DNA was carried out as previously described^39^. To quantify particle-associated HBV DNA, culture supernatants collected from transfected cells were treated with PNE solution (8.45% PEG, 0.445 M NaCl and 13 mM EDTA) for 1 h on ice. To remove free nucleic acids, the pellets were incubated at 37 °C for 1 h with DNase I (TaKaRa, Shiga, Japan) and RNase (TaKaRa). After treatment with proteinase K at 56 °C overnight, HBV DNA was isolated by phenol/chloroform extraction and ethanol precipitation. HBV DNA copies were determined by qPCR with primers 5′-TCCCTCGCCTCGCAGACG-3′ and 5′-GTTTCCCACCTTATGAGTC-3′.

Quantification of unspliced and spliced forms derived from HBV 3.5 kb RNA and host-derived mRNAs were performed as described previously with some modifications^39,40^. Total RNA was extracted from transfected cells with TRI Reagent (Molecular Research Center, Cincinnati, OH, USA). After treatment with DNase I and RNase inhibitor, cDNA templates were synthesized and HBV RNAs were quantified by qPCR using the SYBR qPCR Mix kit (Toyobo, Osaka, Japan) with the following primer sets: 5′-TCCCTCGCCTCGCAGACG-3′ and 5′-GTTTCCCACCTTATGAGTC-3′ for unspliced 3.5 kb RNA, and 5′-CCGCGTCGCAGAAGATCT-3′ and 5′-CTGAGGCCCACTCCCATAGG-3′ for spliced RNAs derived from 3.5 kb RNA. 5′- TTCTACAATGAGCTGCGTGTG -3′ and 5′-GGGGTGTTGAAGGTCTCAAA-3′ for β-actin mRNA, 5′-AAGGGTGTTTCGTTCCCTTT-3′ and 5′-TAGTGTTTTCGCCAAGCAAA-3′ for HSPA1B mRNA, and 5′-AGCAGCAGCTCACCAACC-3′ and 5′-CATCGATTGCAAAGGTGAGA-3′ for PUF60 mRNA. For semi-quantitative RT-PCR, cDNA templates were amplified with primers 5′-AGCCTCCAAGCTGTGCCTTGGGTG-3′ and 5′-AACCACTGAACAAATGGCACTAGTAAACTGAGC-3′. Unspliced and spliced forms of 3.5 kb RNA were analyzed by agarose gel electrophoresis. The nucleotide sequences of PCR primers used in the study are listed in Supplementary Table S1.

### Northern blot analysis

Total RNA was extracted from cells transfected with HBV plasmids using TRI Reagent. After treatment with DNase I and RNase inhibitor, RNA samples were separated on 1.2% agarose gel with 7% formaldehyde at 60 V for 3 h in 1 × 3-(N-morpholino)propanesulfonic acid (MOPS) buffer (20 mM MOPS, 5 mM sodium acetate and 2 mM EDTA). The samples were transferred to a nylon membrane (Roche Diagnostics, Tokyo, Japan) with 20x SSC transfer buffer for 16 h, and subsequently cross-linked to the membrane by ultraviolet light (120 mJ/cm^2^). After washing, the blotted membrane was dried at room temperature. The blot was prehybridized with DIG Easy Hybridization buffer (Roche Diagnostics) in 68 °C and hybridized with an appropriate DIG-labeled RNA probe labeled with DIG-11-UTP at 68 °C overnight using the DIG Northern Starter Kit (Roche Diagnostics). To generate a DIG-labeled RNA probe with specific binding to HBV pregenome and HBs RNA, PCR fragments covering the nt 1998–2447 and nt 3205–488 regions were used as templates for *in vitro* transcription for the pregenome probe and HBs probe, respectively. RNA was labeled in the T7 promoter transcriptional system with DIG-11-UTP using a labeling mixture from the DIG Northern Starter Kit (Roche Diagnostics). Detection of the DIG-labeled probe on the blot was performed using CDP-Star detection reagent (GE Healthcare, Tokyo, Japan).

### RNA degradation assay

At day 2 post-transfection (pt) with pUC-HB-Ce and pcDNA-F-PUF60 or pcDNA-F-PUF60-D1, aliquots of the cells were harvested (designated as 0 h) and the other cells were treated with actinomycin D (5 µg/ml), followed by further culture for 6 or 12 h. At each time point, total RNA was extracted from transfected cells with TRI Reagent and the HBV 3.5 kb RNA level was determined by RT-qPCR using the SYBR qPCR Mix kit (Toyobo).

### Luciferase reporter assay

Cells were transiently co-transfected with pcDNA-F-PUF60, pcDNA-F-PUF60-D1, -D2, -D3 or empty vector and the *Renilla* luciferase reporter which carries either of HBV promoter or host cellular promoter. At 24 or 48 h pt, luciferase activities in cell lysates were measured with the *Renilla* luciferase reporter assay kit (Promega). Total protein concentrations in cell lysates were measured and used to normalize luciferase activities.

### Gel mobility shift assay

To determine *in vitro* binding between transcriptional factors and HBV DNA sequence of the ENII/BCP region, HNF1α, SRY, TCF7L2, FOXM1, SP1, KLF5 and PUF60 were synthesized *in vitro*. In brief, cDNAs encoding these seven transcription factors were isolated from MGC clones (DNAFORM, Yokohama, Japan) and individually inserted into a pEU vector^41^ to express an N-terminal FLAG-fusion protein. Each transcription factor was synthesized in a wheat cell-free system as previously described^42^. The 3′-ends of synthesized oligonucleotides (nt 1689–1726) were labeled by DIG-11-dUTP using the DIG gel shift kit (Roche Diagnostics). The labeled oligonucleotide probe was mixed with each synthesized protein, and the gel shift reaction was performed according to the manufacturer’s instructions. The resulting samples were analyzed by native PAGE using a 6% gel. The labeled DNA-protein complexes as well as the probe were blotted to a nylon membrane and detected using CDP-Star detection reagent (GE Healthcare, Buckinghamshire, UK).

### Immunoblotting and immunocytochemistry

Immunoblotting was performed as previously described with slight modification^43^. Briefly, cell lysates were separated by SDS–PAGE and transferred onto polyvinylidene difluoride membranes. After blocking, membranes were incubated with an antibody against PUF60 (GeneTex, Irvine, CA), FLAG M2 (Sigma-Aldrich, Tokyo, Japan) or GAPDH (Santa Cruz Biotechnology, Dallas, TX) or HA (MBL, Nagoya, Japan) for 1 h. After washing, membranes were incubated with an HRP-conjugated secondary antibody (Cell Signaling Technology, Danvers, MA) for 0.5–1 h. Antigen-antibody complexes were detected using the ChemiDoc™ Imaging System (BIO-RAD Laboratories, Tokyo, Japan). For immunocytochemistry, cells grown on a glass bottom plate were fixed with 4% paraformaldehyde for 15 min and permeabilized in 0.5% Triton X-100 in PBS, followed by blocking with 1% bovine serum albumin (BSA). Immunocytochemistry was performed by incubation with the anti-PUF60 antibody (GeneTex, Irvine, CA) for 2 h, followed by incubation with Alexa Fluor 488 anti-rabbit IgG (H + L) antibody (Vector Laboratories, Burlingame, CA, USA) for 2 h. Double-stranded DNA was stained with Hoechst 33342 (Dojin, Tokyo, Japan). Subcellular localization of PUF60 was observed under a confocal microscope (Leica TCS SP8; Leica, Wetzlar, Germany).

### ChIP assay

ChIP followed by qPCR was performed as previously described with some modification^44^. Briefly, cells seeded in 100-mm dishes were transfected with siTCF7L2 RNA. After 48 h, the cells were co-transfected with pUC-HB-Ce and the pcDNA-F-PUF60 expression vector or empty vector. After 48 h, ChIP was performed by the Chromatin IP kit (Cell Signaling Technology). Proteins in the cells were cross-linked with DNA using 1% formaldehyde for 10 min at room temperature. The cross-linking reaction was stopped by the addition of 1 ml of 10x glycine to each dish and incubation for 5 min at room temperature. After washing two times with ice-cold PBS, the cells were scraped into PIC buffer (1 ml PBS and 5 µl 200x protease inhibitor cocktail) and sonicated to shear DNA to lengths between 150 and 900 bp. After 5-fold dilution of the sonicated cell supernatants in 100 µl 1x ChIP buffer and 0.5 µl 200x PIC, immunoprecipitations were carried out overnight at 4 °C with the anti-FLAG M2 antibody. Protein G agarose beads were added and incubated for 2 h at 4 °C with rotation. DNA-protein complexes were eluted from the beads with a buffer containing 1% SDS and 0.1 M NaHCO_3_. The cross-links were reversed by incubating the eluates with proteinase K solution (final concentration: 200 mM NaCl and 266 µg/ml proteinase K) overnight at 65 °C. DNA was recovered by phenol/chloroform extraction and ethanol precipitation. ChIPped DNA was analyzed for the presence of HBV gene promoter sequence by qPCR. Viral DNA covering the nt 1589–1828 region was detected by using the SYBR qPCR Mix kit (Toyobo) with the following primer set: 5′-CTTCACCTCTGCACGTCGCATG-3′ and 5′-GTGAAAAAGTTGCATGGTGCTGGTG-3′.

### Immunoprecipitation

Immunoprecipitation was performed as previously described with slight modification^14^. Briefly, cells were lysed with lysis buffer (0.5% NP-40 in PBS) and centrifuged at 15,000 rpm for 10 min at 4 °C. The supernatants were incubated with Protein G agarose beads, which were prewashed with lysis buffer, and anti-HA antibody or anti-FLAG antibody for 60 min at 4 °C. The samples were then centrifuged, and the resulting pellets were washed 4 times with lysis buffer and subjected to SDS-PAGE.

### Subcellular fractionation

Cells were suspended with hypotonic buffer (0.5% NP40,10 mM Tris-HCl pH8.0, 10 mM NaCl, 3 mM MgCl2, 5 mM DTT), followed by centrifugation at 500 × g for 5 min at 4 °C. The supernatant was collected and termed as the cytoplasmic fraction. The pellet containing the nuclear fraction was re-suspended with disruption buffer (1% Triton, 1% DOC, 0.1% SDS, 25 mM Tris-HCl pH 7.6, 150 mM NaCl, 1 mM EDTA, 5 mM DTT).

## Electronic supplementary material

Supplementary Information
