# Supplementary material for: Involvement of PUF60 in Transcriptional and Post-transcriptional Regulation of Hepatitis B Virus Pregenomic RNA Expression
Source: Sci Rep. 2017 Oct 9;7:12874. doi: 10.1038/s41598-017-12497-y (PMC5634508; doi:10.1038/s41598-017-12497-y)

Involvement of PUF60 in Transcriptional and Post-transcriptional Regulation of Hepatitis B Virus Pregenomic RNA Expression

Suofeng Sun,[1] Kenji Nakashima,[1] Masahiko Ito,[1] Yuan Li,[1] Takeshi Chida,[1] Hirotaka Takahashi,[2] Koichi Watashi,[3] Tatsuya Sawasaki,[2] Takaji Wakita,[3] Tetsuro Suzuki [1*]

[1] Department of Virology and Parasitology, Hamamatsu University School of Medicine, Shizuoka 431-3192, Japan

[2] Proteo-Science Center, Ehime University, Ehime, 790-8577, Japan

[3] Department of Virology II, National Institute of Infectious Diseases, Tokyo 162-8640, Japan

Supplementary methods

Chemiluminescent immunoassay

The quantitative determination of HBs in supernatant was performed by LSI Medience (Tokyo, Japan) using the Architect HBsAg QT assay (Abbott, Chicago, IL).

Immunoblotting

Cell lysates were separated by SDS–PAGE and transferred onto polyvinylidene difluoride membranes. After blocking, membranes were incubated with an antibody against HBs (Institute of Immunology, Tokyo, Japan), HBc (described previously[1]), GAPDH (Santa Cruz Biotechnology, Dallas, TX), LMNB1 (GeneTex, Irvine, CA), PUF60 (GeneTex), or HA (MBL, Nagoya, Japan) for 1 h. After washing, membranes were incubated with an HRP-conjugated secondary antibody (Cell Signaling Technology, Danvers, MA) for 0.5–1 h. Antigen-antibody complexes were detected using the ChemiDoc™ Imaging System (BIO-RAD Laboratories, Tokyo, Japan).

Amplified luminescent proximity homogeneous assay

Biotinylated RNA fragments corresponding to HBV PRE (nt 1225-1585) and reverse PRE were synthesized using the MEGAscript T7 kit (Thermo Fisher Scientific). FLAG-tagged PUF60 was synthesized in vitro with pEU-E01-PUF60 as the template by using the WEPRO *in vitro* translation kit (CellFree Sciences, Ehime, Japan). RNA-PUF60 binding assays were performed in a final reaction volume of 25 µl containing 100 ng, 200 ng or 400 ng of biotinylated RNA, 0.1% BSA (w/v), 40 U RNase inhibitor, 20 µg/ml anti-FLAG (M2) acceptor beads, 20 µg/ml streptavidin donor beads and 2.5 µl of 10x AlphaScreen assay buffer (PerkinElmer, Waltham, MA). After incubation for 60 min at 25°C, the reaction samples were read using an EnSpire plate reader (PerkinElmer) to determine AlphaScreen signals (photon counts at 630 nm/s).

Electroporation of in vitro synthesized HBV RNA

pBS-based plasmid carrying the 2-fold HBV genome[2] was digested with SacI and was used as a template for RNA synthesis. An around 6-kb HBV RNA, started at nt 128, was synthesized *in vitro* by use of a mMESSAGE mMACHINE T7 Transcription Kit (Thermo Fisher Scientific). A poly(A) tail was added to the transcripts using *E. coli* Poly(A) Polymerase (Thermo Fisher Scientific). Trypsinized cells were washed with phosphate buffered saline (PBS) and resuspended at 1 x 10^5 cells/10 μL with BTXpress buffer (BTX, Holliston, MA). 0.3μg of HBV RNA was mixed with 10 μL of cell suspension and electroporated by Neon (Thermo Fisher Scientific). The condition of electroporation was at 1200 V, 20 msec and 1 pulse. Resulting cells were immediately seeded into 24-well plates.

Supplemental reference

1 Li, Y. et al. LUC7L3/CROP inhibits replication of hepatitis B virus via suppressing enhancer II/basal core promoter activity. *Sci Rep* **6**, 36741, doi:10.1038/srep36741 (2016).

2 Fujiyama, A. et al. Cloning and structural analyses of hepatitis B virus DNA, subtype adr. *Nucleic Acids Res* **11**, 4601–4610 (1983)

Supplementary Table S1

Nucleotide sequences of PCR primers used in this study

PCR primers for particle-associated HBV DNA

Primer Name	sequence (5'-3')
HBV DNA forward	TCCCTCGCCTCGCAGACG
HBV DNA reverse	GTTTCCCACCTTATGAGTC

Primers for RT-qPCR

Primer Name	sequence (5'-3')
3.5 kb RNA forward	TCCCTCGCCTCGCAGACG
3.5 kb RNA reverse	GTTTCCCACCTTATGAGTC
spliced RNA forward	CCGCGTCGCAGAAGATCT
spliced RNA reverse	CTGAGGCCCACTCCCATAGG
β-actin mRNA forward	TTCTACAATGAGCTGCGTGTG
β-actin mRNA reverse	GGGGTGTTGAAGGTCTCAAA
PUF60 mRNA forward	AGCAGCAGCTCACCAACC
PUF60 mRNA reverse	CATCGATTGCAAAGGTGAGA
HSPA1B mRNA forward	AAGGGTGTTTCGTTCCCTTT
HSPA1B mRNA reverse	TAGTGTTTTCGCCAAGCAAA

Primers for semi-quantitative RT-PCR

Primer Name	sequence (5'-3')
HBV RNA forward	AGCCTCCAAGCTGTGCCTTGGGTG
HBV RNA reverse	AACCACTGAACAAATGGCACTAGTAAACTGAGC

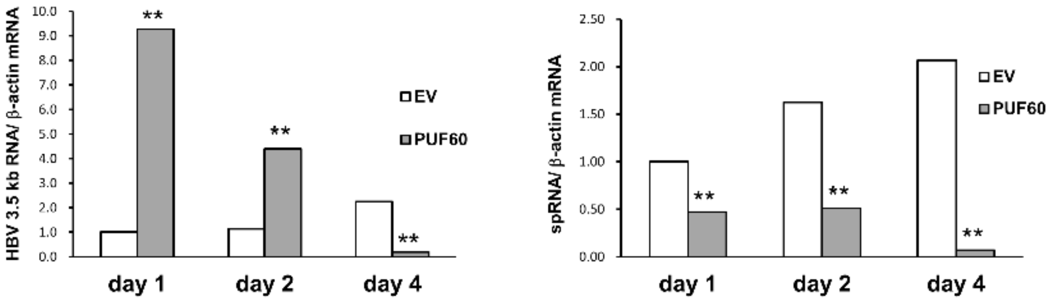

Supplementary Figure S1

Time-course changes of HBV 3.5 kb RNA and spliced forms derived from 3.5 kb RNA (spRNA). pcDNA-F-PUF60 or an empty vector (EV) was co-transfected with pUC-HB-Ce carrying the 1.24-fold HBV genome into HuH-7 cells. At day 1,2 or 4 post-transfection (pt), total RNA was extracted from cells. RT-qPCR analysis was carried out to determine 3.5 kb RNA and spliced RNA (spRNA) levels. Data are normalized to that of β-actin mRNA and the values in cells transfected with EV at 1 pt are set to 1. Values shown represent means ± SD obtained from three independent samples. Statistical differences compared with the control (EV) are shown. **$p<0.01$, Student's t test.

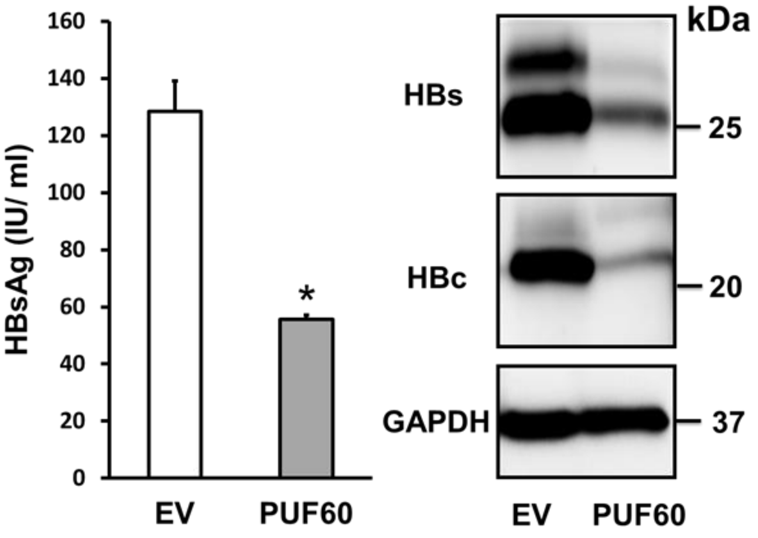

Supplementary Figure S2

Effect of PUF60 expression on expression of HBV proteins. pcDNA-F-PUF60 or an empty vector (EV) was co-transfected with pUC-HB-Ce carrying the 1.24-fold HBV genome into HuH-7 cells. At day 4 pt, the culture supernatants of the cells were collected and analyzed by chemiluminescent immunoassay to determine HBs antigen (left). The cells were lysed and used for immunoblotting with anti-HBs, anti-HBc or anti-GAPDH antibodies (right).

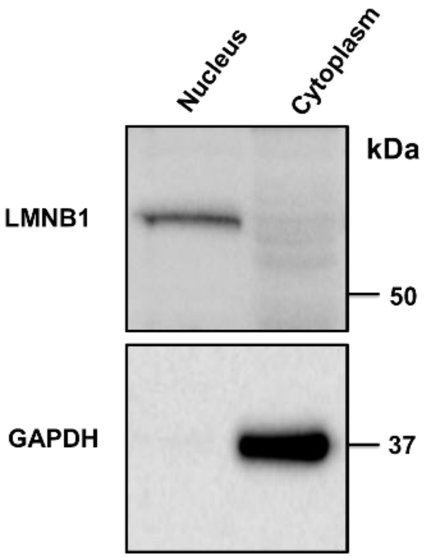

Supplementary Figure S3

Detection of marker proteins for nucleus and cytoplasm in isolated fractions. Nuclear and cytoplasmic fractions from HuH-7 cell lysates were prepared and were subjected to immunoblotting to detect Lamin B1 (LMNB1) and GAPDH, as the nuclear and cytoplasmic markers, respectively.

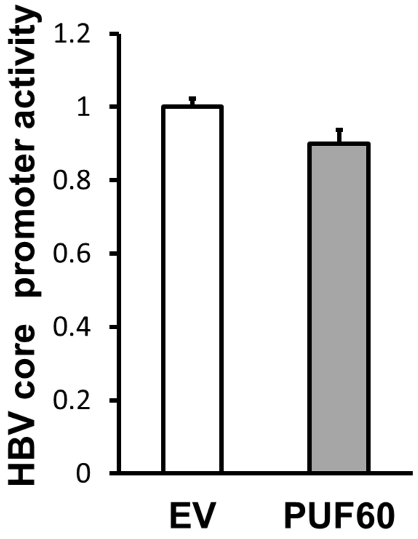

Supplementary Figure S4

Effect of PUF60 expression on core promoter at day 4 pt. HuH-7 cells were transfected with the luciferase reporter carrying the entire core promoter (nt 900-1817) and pcDNA-F-PUF60 or empty vector (EV). At day 4 pt, reporter activities in the cells were measured. Values are normalized to total protein concentrations in cell lysates and the resulting value in cells transfected with EV was set to 1. This assay was performed in triplicate and the results are presented as means ± SD.

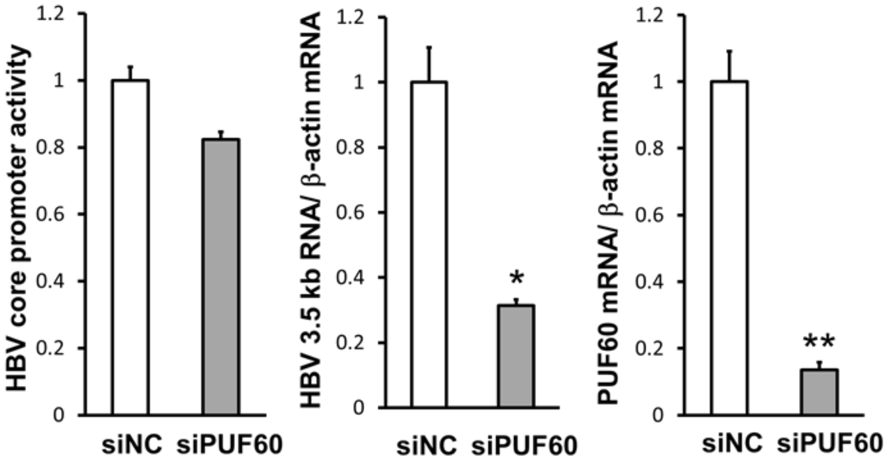

Supplementary Figure S5

Knockdown effect of PUF60 on core promoter activity and 3.5 kb RNA expression. At 4 days after introducing PUF60 siRNAs (siPUF60) or its negative control (siNC), HuH-7 cells were transfected with pGLHBp900/1817 or pUC-HB-Ce and then reporter activities and RNA levels, respectively, were measured after 2 days of further culture. PUF60 mRNA expression was also determined. Data were normalized to that of β-actin mRNA and the values in cells transfected with EV or siNC were set to 1. All assays were performed in triplicate and results are presented as means ± SD. Statistical differences compared with the control (EV or siNC) are shown. *$p<0.05$, **$p<0.01$, Student’s t test.

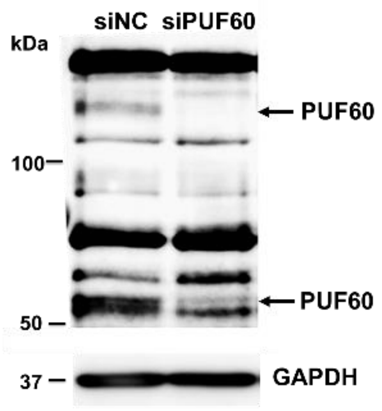

Supplementary Figure S6

Immunoblotting to indicate PUF60 gene knockdown by siRNA. HuH-7 cells were transfected with siRNA for PUF60 (siPUF60) or its negative control (siNC). At 2 days post-transfection, the cells were lysed and separated by SDS–PAGE, followed by immunoblotting with anti-PUF60 or anti-GAPDH antibody. Closed arrow heads represent PUF60.

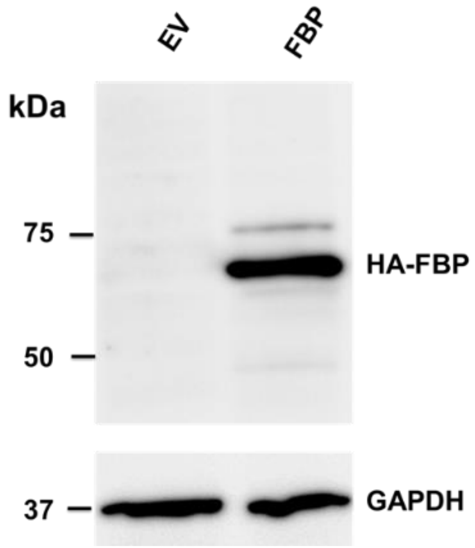

Supplementary Figure S7

Expression of HA-tagged FBP protein from pcDNA-HA-FBP was confirmed. At day 2 pt with pcDNA-HA-FBP (FBP) or an empty vector (EV), HuH-7 cells were lysed and separated by SDS–PAGE, followed by immunoblotting with anti-HA or anti-GAPDH antibodies.

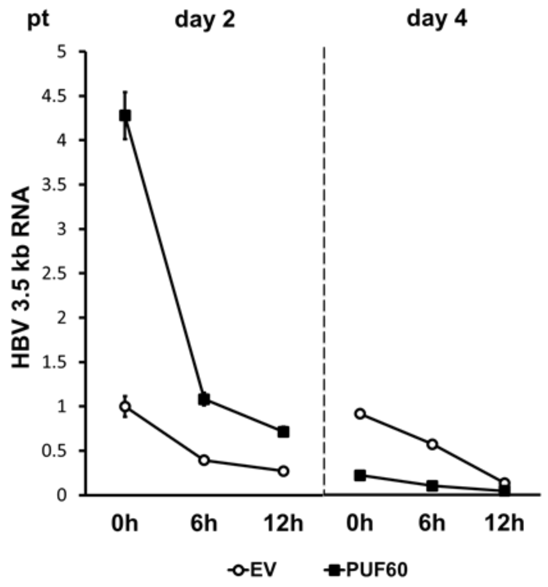

Supplementary Figure S8

Involvement of PUF60 in HBV 3.5 kb RNA degradation. Time course change in HBV 3.5 kb RNA levels was determined. At day 2 or 4 pt with pUC-HB-Ce and pcDNA-F-PUF60 or empty vector (EV), aliquots of cells were harvested (designated as 0 h) and the other cell settings were treated with actinomycin D (5 µg/ml), followed by further culture for 6 or 12 h. At each time point, total RNA was extracted and HBV 3.5 kb RNA levels were assessed by RT-qPCR. The values in cells transfected with EV at 0 h were set to 1 in each panel. Assays were performed in triplicate and results are presented as means ± SD. It is noted that measured data for EV control and PUF60 expression at day 2 pt were also used in Fig 7a where the values of each transfection group at 0 h were set to 1.

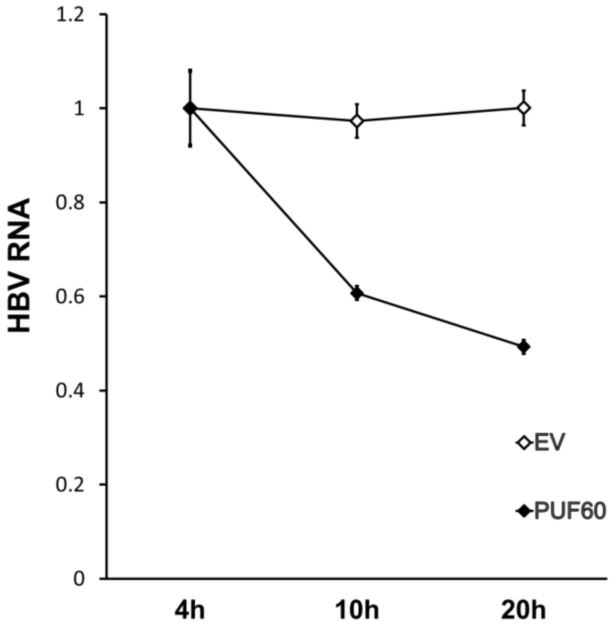

Supplementary Figure S9

In vitro synthesized HBV RNA was electroporated into cells after 4 days of the culture that was transfected either with pcDNA-F-PUF60 or an empty vector (EV), followed by monitoring the viral RNA level at 4 h, 10 h and 20 h after the RNA electroporation. At each time point, aliquots of cells were harvested and total RNA was extracted. The HBV RNA levels were then assessed by RT-qPCR. The values of each group at 4h were set to 1. Assays were performed in triplicate and results are presented as means ± SD.

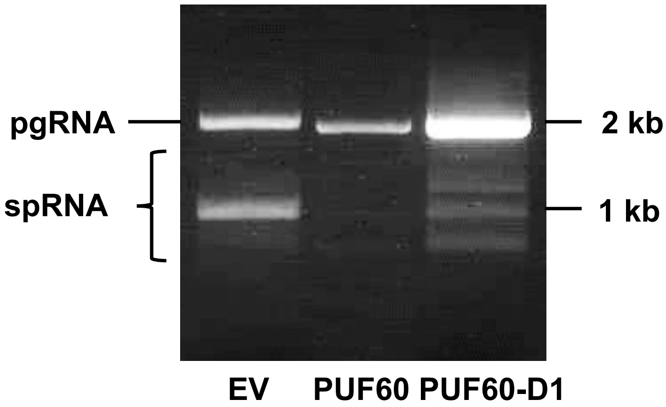

Supplementary Figure S10

Effects of PUF60 and PUF60-D1 on 3.5 kb RNA splicing. Cells were transfected with pUC-HB-Ce and pcDNA-F-PUF60, -PUF60-D1 or EV, followed by semi-quantitative RT-PCR at day 4 pt.

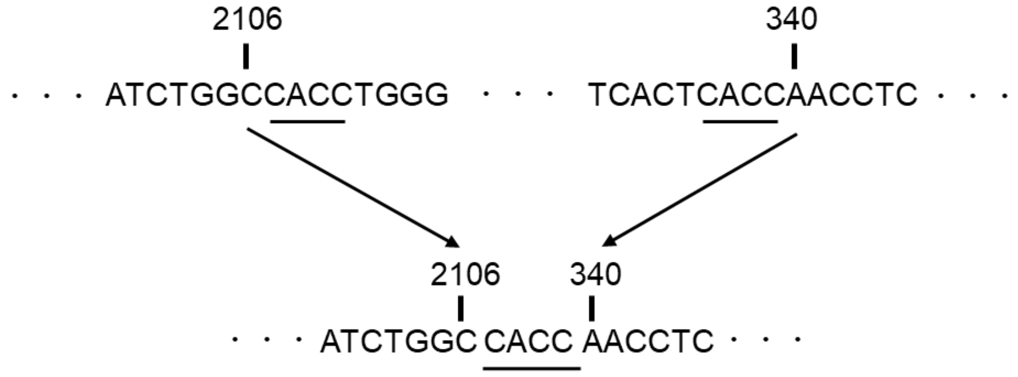

Supplementary Figure S11

cDNA sequence of the junction site in the alternative splicing (indicated as an asterisk in Fig. 7c, right) derived from 3.5 kb HBV RNA induced by PUF60 expression. Homologous sequences conserved at nt 2107-2110 and 336-339 were shown with underlines.

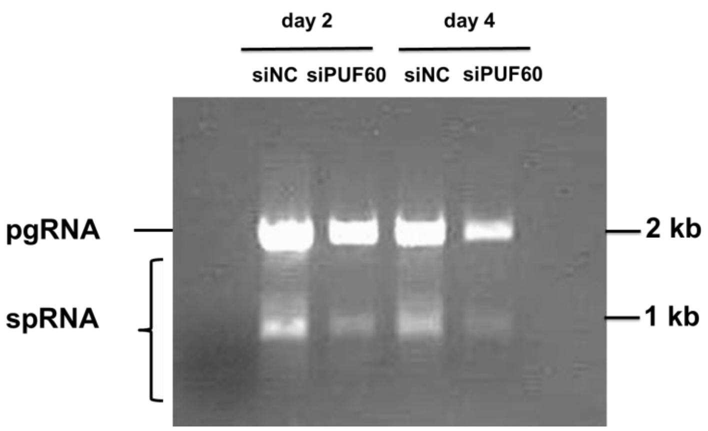

Supplementary Figure S12

Effect of PUF60 knockdown on the HBV RNAs at day 2 and 4 pt. At 2 days after introducing PUF60 siRNAs (siPUF60) or its negative control (siNC), HuH-7 cells were transfected with pUC-HB-Ce and then HBV RNAs were determined by semi-quantitative RT-PCR after 2 and 4 days of further culture.

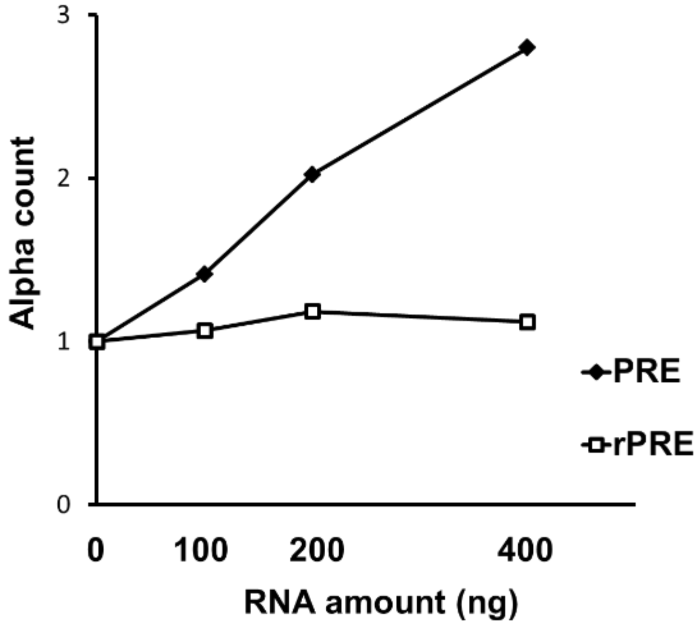

Supplementary Figure S13

Physical interaction of PUF60 with post-transcriptional regulatory element (PRE) of HBV RNA. The interaction between Flag-tagged PUF60 and biotinylated PRE (nt 1225-1585) or its reverse sequence (rPRE) was determined by the *in vitro* amplified luminescent proximity homogeneous assay.

Supplementary Figure S14

Full-length blots used to generate Fig. 1a

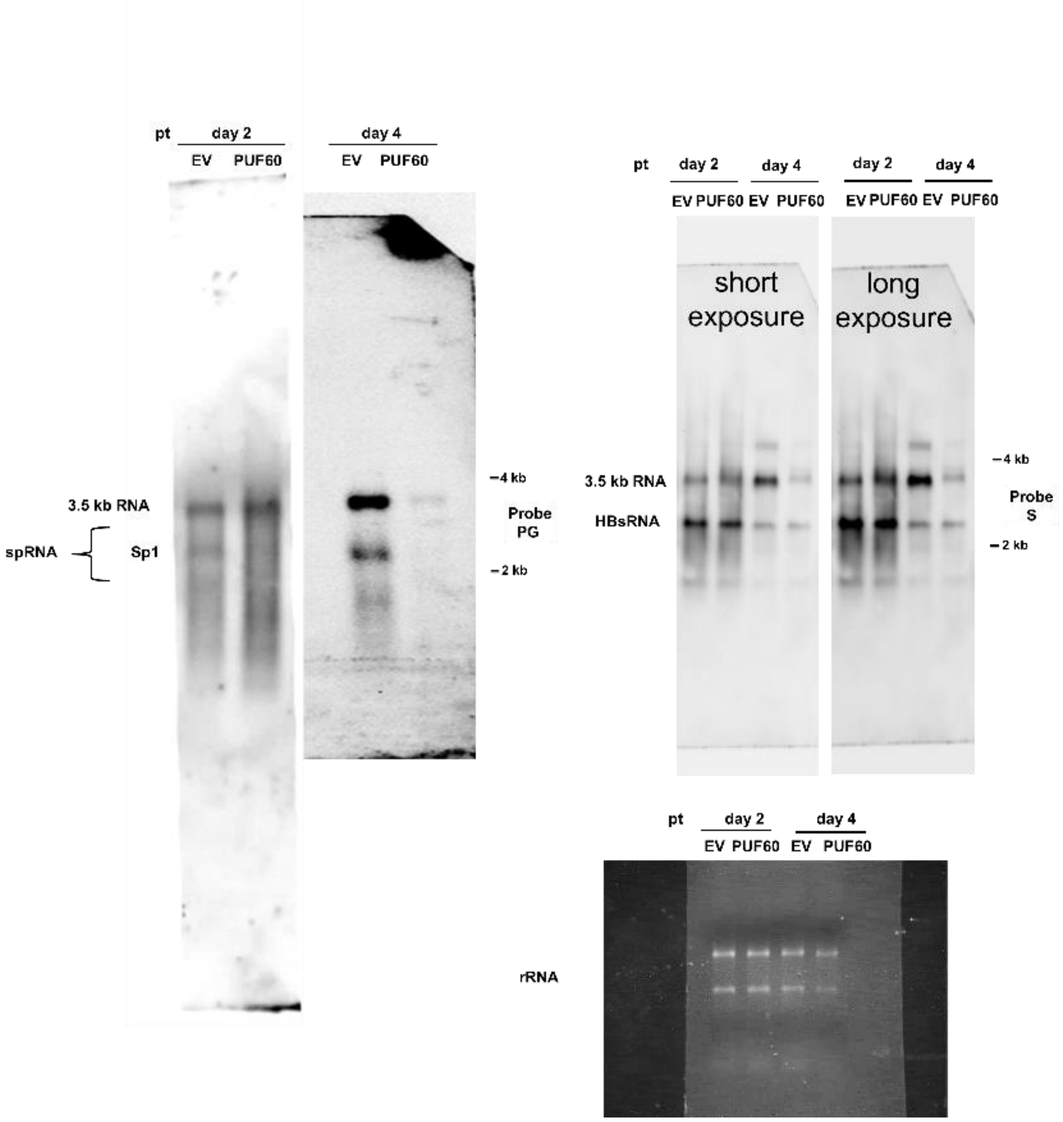

Supplementary Figure S15

Full-length blots used to generate Fig. 1b

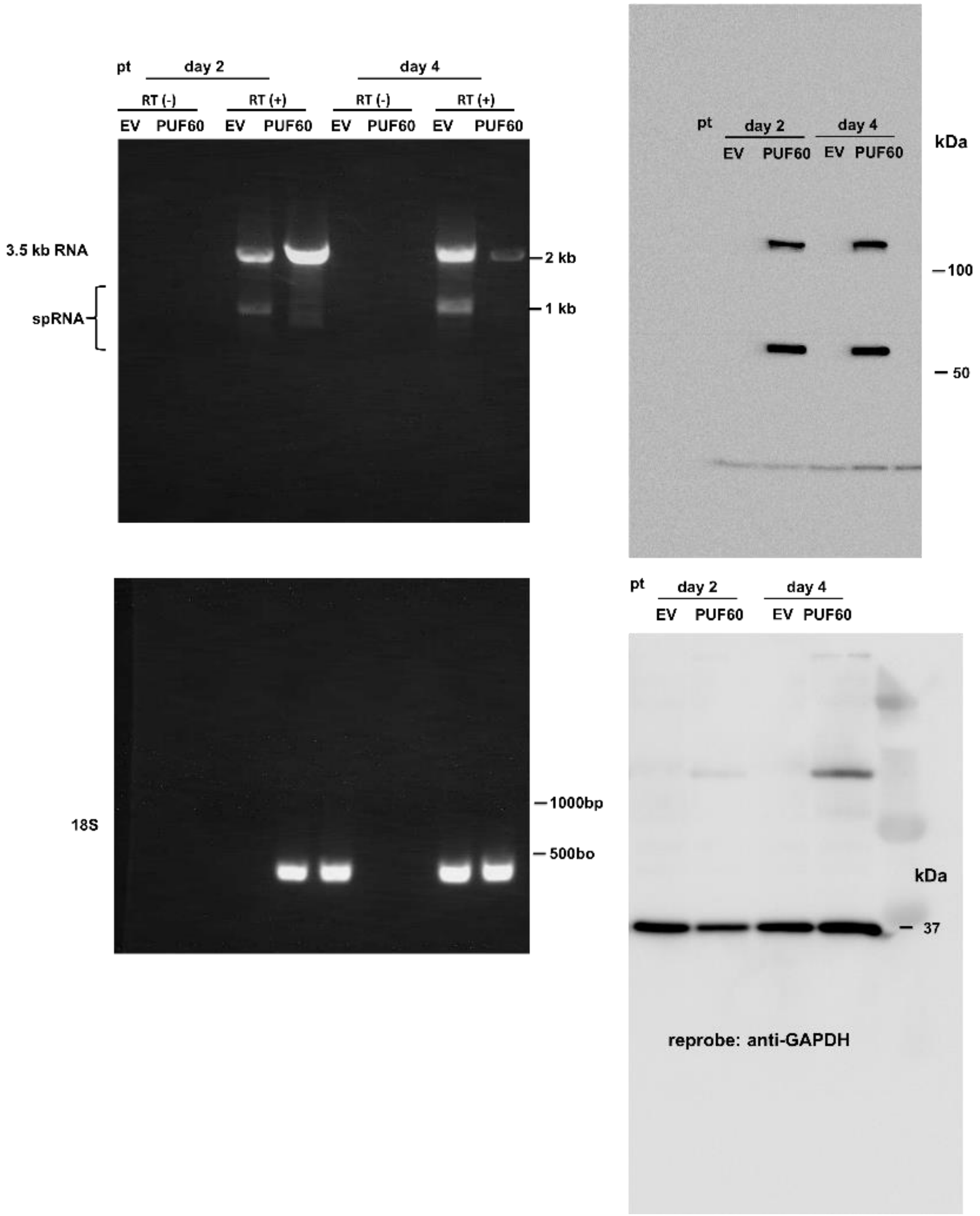

Supplementary Figure S16

Full-length blot used to generate Fig. 2c

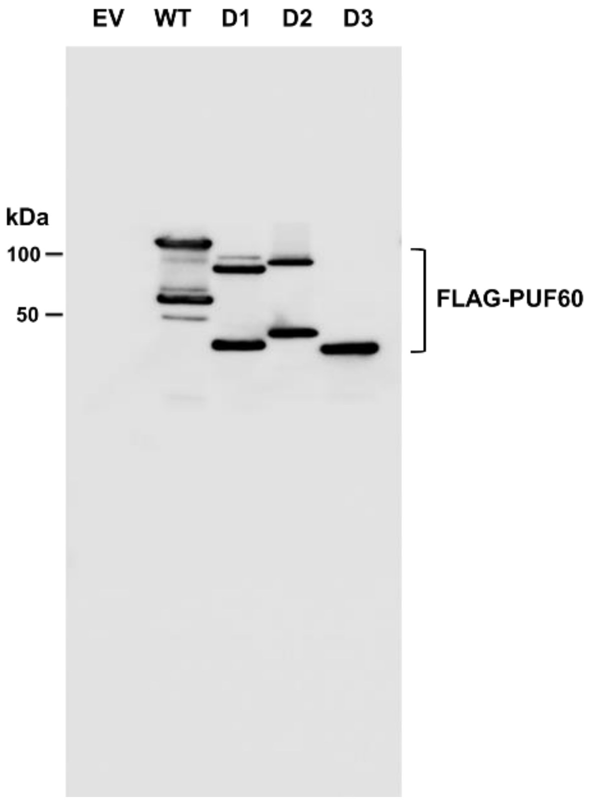

Supplementary Figure S17

Full-length blots used to generate Fig. 6b

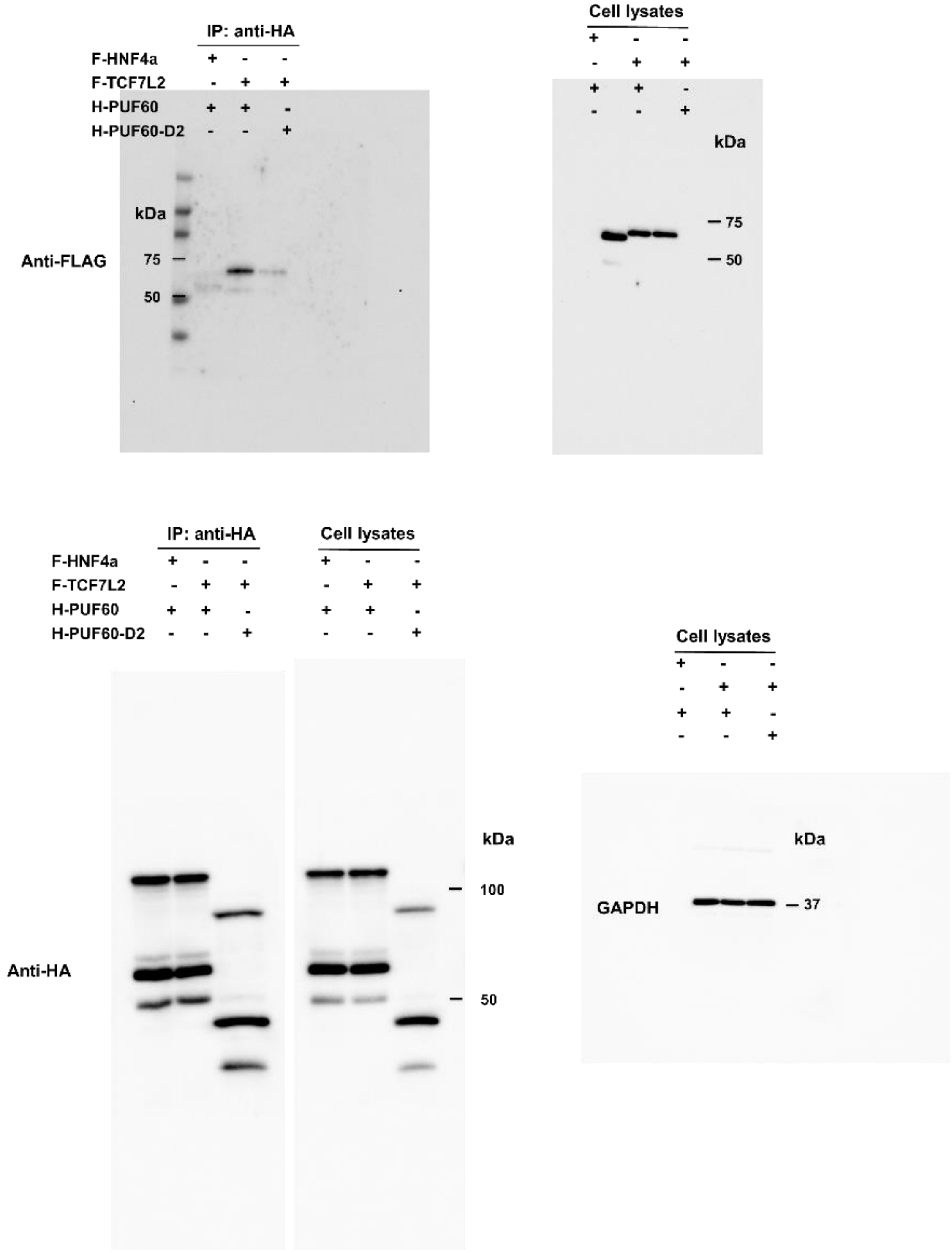

Supplementary Figure S18
Full-length blots used to generate Fig. 6c

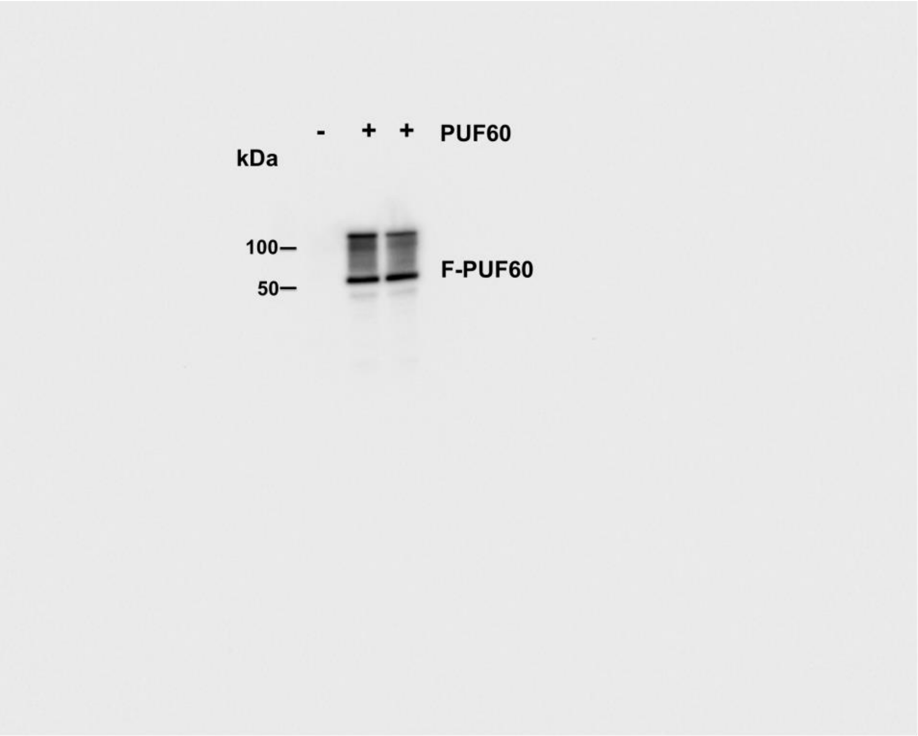

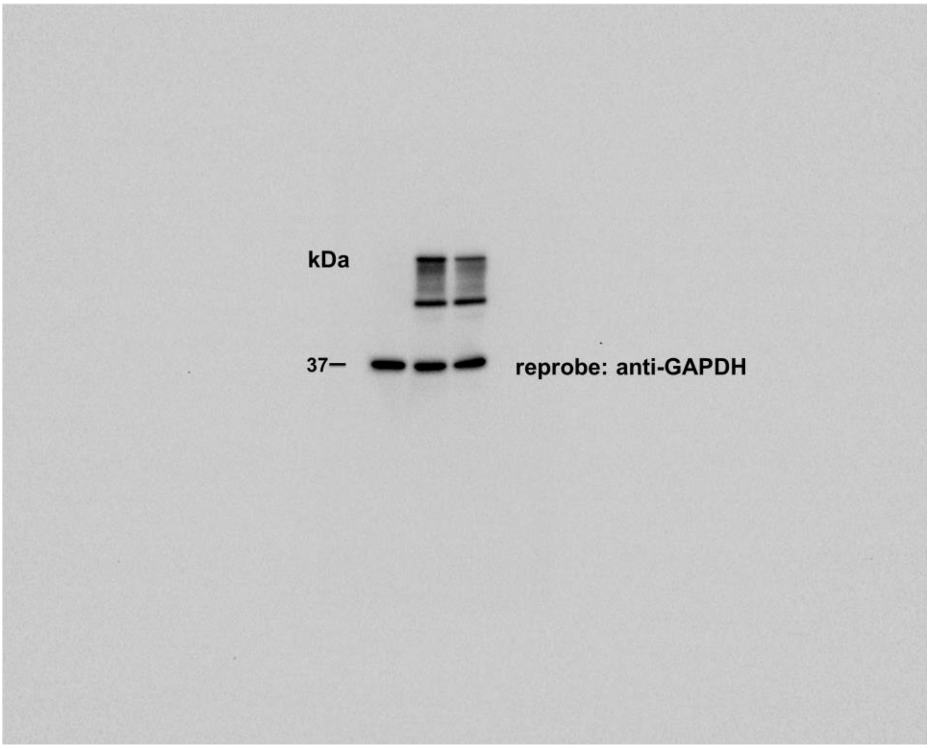

Supplementary Figure S19

Full-length blots used to generate Fig. 7a

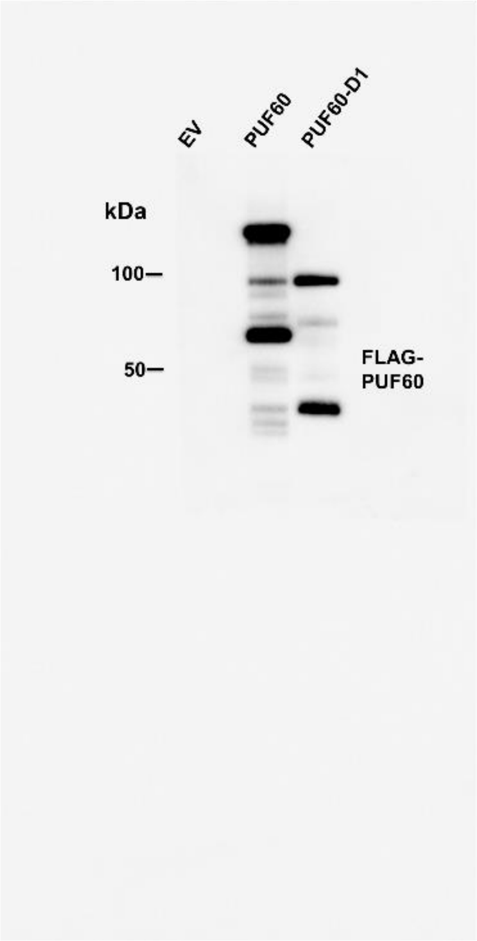

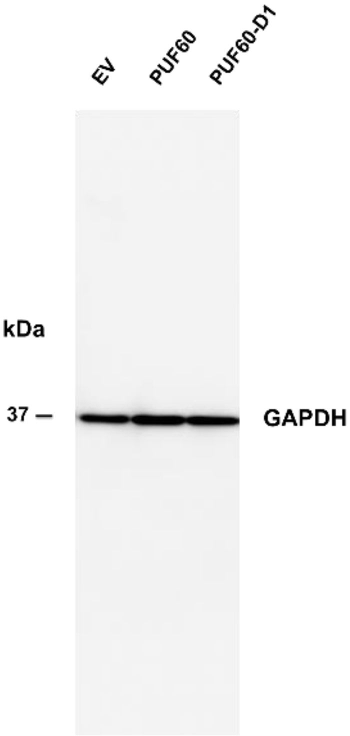

Supplementary Figure S20

Full-length blots used to generate Fig. 7b

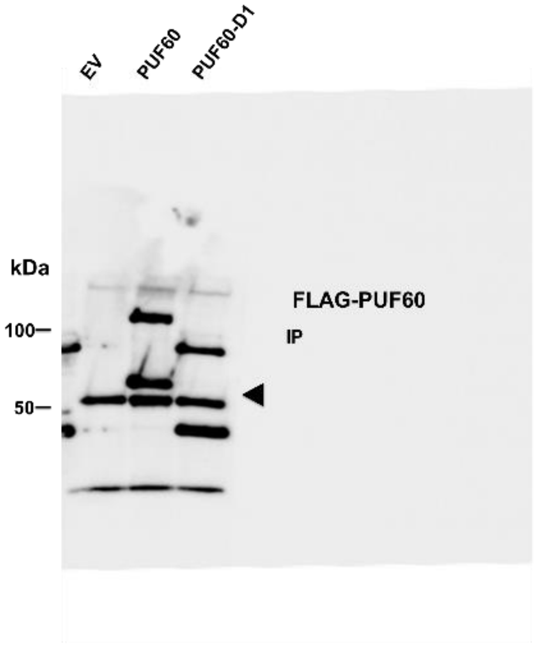

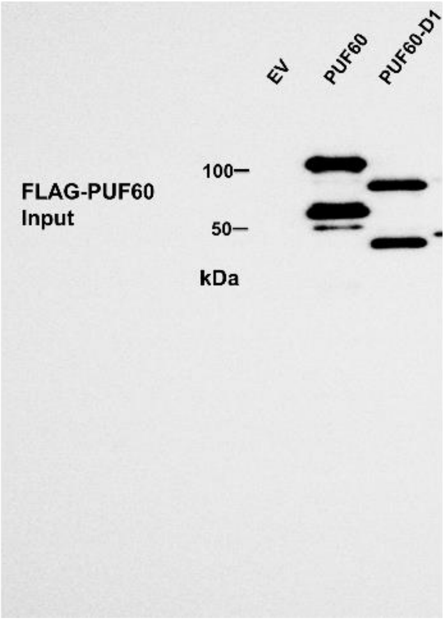

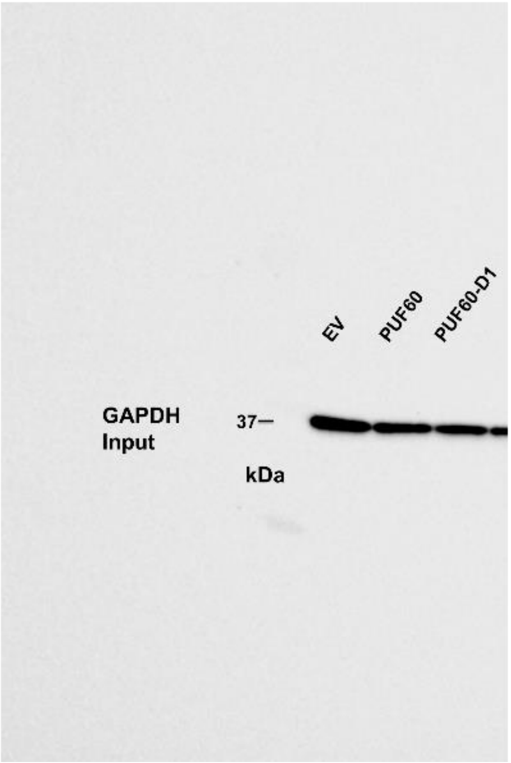

Supplementary Figure S21
Full-length blot used to generate Fig. 7c

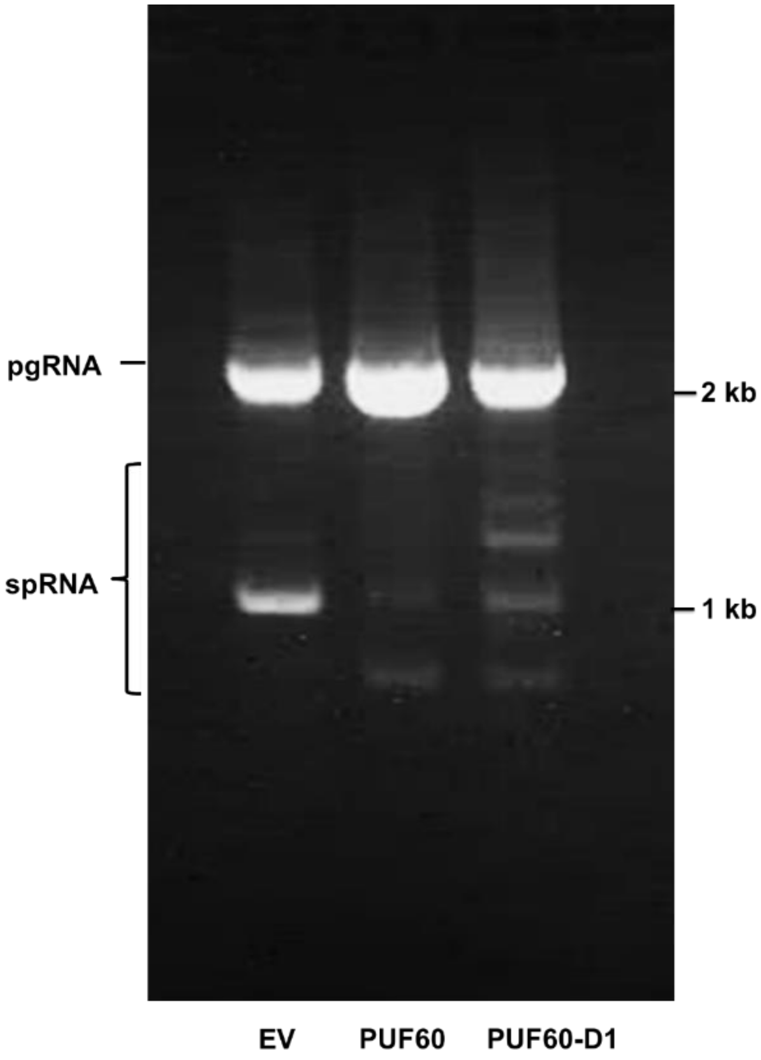

Supplement: Supplementary file 1 — Supplementary Information [file 41598_2017_12497_MOESM1_ESM.pdf]
